# Glutamine is essential for overcoming the immunosuppressive microenvironment in malignant salivary gland tumors

**DOI:** 10.7150/thno.73896

**Published:** 2022-08-08

**Authors:** Shuting Cao, Yu-Wen Hung, Yi-Chang Wang, Yiyin Chung, Yue Qi, Ching Ouyang, Xiancai Zhong, Weidong Hu, Alaysia Coblentz, Ellie Maghami, Zuoming Sun, H. Helen Lin, David K. Ann

**Affiliations:** 1 Department of Diabetes Complications and Metabolism, Arthur Riggs Diabetes and Metabolism Research Institute, Beckman Research Institute, City of Hope, Duarte, CA, 91010, USA; 2 Department of Computational and Quantitative Medicine, Beckman Research Institute, City of Hope Comprehensive Cancer Center, Duarte, CA 91010, USA; 3 Department of Immunology and Theranostics, Beckman Research Institute, City of Hope Comprehensive Cancer Center, Duarte, CA 91010, USA; 4 Division of Head and Neck Surgery, City of Hope National Medical Center, Duarte, CA 91010, USA; 5 Irell & Manella Graduate School of Biological Sciences, Beckman Research Institute, City of Hope Comprehensive Cancer Center, Duarte, CA 91010, USA

**Keywords:** autophagy, tumor microenvironment, glutamine, CD8, Treg

## Abstract

**Rationale:** Immunosuppression in the tumor microenvironment (TME) is key to the pathogenesis of solid tumors. Tumor cell-intrinsic autophagy is critical for sustaining both tumor cell metabolism and survival. However, the role of autophagy in the host immune system that allows cancer cells to escape immune destruction remains poorly understood. Here, we determined if attenuated host autophagy is sufficient to induce tumor rejection through reinforced adaptive immunity. Furthermore, we determined whether dietary glutamine supplementation, mimicking attenuated host autophagy, is capable of promoting antitumor immunity.

**Methods:** A syngeneic orthotopic tumor model in *Atg5^+/+^* and *Atg5^flox/flox^* mice was established to determine the impact of host autophagy on the antitumor effects against mouse malignant salivary gland tumors (MSTs). Multiple cohorts of immunocompetent mice were used for oncoimmunology studies, including inflammatory cytokine levels, macrophage, CD4^+^, and CD8^+^ cells tumor infiltration at 14 days and 28 days after MST inoculation. *In vitro* differentiation and *in vivo* dietary glutamine supplementation were used to assess the effects of glutamine on Treg differentiation and tumor expansion.

**Results:** We showed that mice deficient in the essential autophagy gene, *Atg5*, rejected orthotopic allografts of isogenic MST cells. An enhanced antitumor immune response evidenced by reduction of both M1 and M2 macrophages, increased infiltration of CD8^+^ T cells, elevated IFN-γ production, as well as decreased inhibitory Tregs within TME and spleens of tumor-bearing *Atg5^flox/flox^* mice. Mechanistically, ATG5 deficiency increased glutamine level in tumors. We further demonstrated that dietary glutamine supplementation partially increased glutamine levels and restored potent antitumor responses in *Atg5^+/+^* mice.

**Conclusions:** Dietary glutamine supplementation exposes a previously undefined difference in plasticity between cancer cells, cytotoxic CD8^+^ T cells and Tregs.

## Introduction

Accumulating evidence suggests that the modulation of immune microenvironment plays a critical role in anti-cancer immunity by regulating both tumor immune surveillance and evasion 1-3. Notably, the immunosuppressive networks promote cancer progression, metastasis and resistance to therapies 1. Salivary gland tumors have more than 30 subtypes, among them, salivary duct carcinoma (SDC), albeit rare, represents the most lethal and aggressive histologic subtype 4. A recent study by Linxweiler et al. compared the immune landscape of malignant salivary gland tumors (MSTs) and revealed a significantly higher overall immune score 5. In addition, several groups reported that MSTs exhibit higher levels of cytotoxic T lymphocyte (CTL) dysfunction (epitomized by overexpression of immune checkpoint genes) and an abundance of immune-suppressive cell types (exemplified by regulatory T cells [Tregs]) 6-10. Thus, MSTs have evolved strategies to module the immune microenvironment to evade antitumor immune response.

Accumulating evidence has suggested the involvement of the various nutrients in regulating the survival, apoptosis, differentiation, activation, effector function and tumor trafficking of immune cell subsets 11-13. For example, during T-cell differentiation, CD4^+^ and CD8^+^ cells are generated from naïve T cells through distinct glucose-mediated activation of effector function and clonal selection 12. CD8^+^ T cells are the preferred immune cells for targeting cancer cells for immunogenic cell death 14, 15. In parallel, the naïve T cells, activated by antigen-presenting cells and specific cytokines, differentiate into CD4^+^ effector cells (T helper cells and Th17 cells) as well as Tregs 11, 16. Notably, Treg cells exhibited increased fatty acid oxidation, whereas Th17 cells have demonstrated a reliance upon fatty acid synthesis 17. Tregs appear to play a major role in suppressing antitumor immune responses 18. The precise nutrient utilization pathways regulating Treg functions and the crosstalk between different T lymphocyte subsets to govern antitumor immunity remains unclear.

Autophagy recycles cargos to provide anabolic and catabolic substrates 19. This metabolic recycling function of autophagy promotes tumor cell survival under conditions of nutrient limitation 20. Furthermore, autophagy may favor tumor progression by promoting the escape of malignant cells from immune surveillance 21-25. Autophagosome formation during autophagy involves various autophagy-related genes (Atgs), including *Atg5* 26. Indeed, elevated *Atg5* expression is an unfavorable prognostic marker for human renal and hepatic cancers (The Human Protein Atlas; 27). Moreover, autophagy plays a key role in shaping T cell immunity and activation 28, 29. During the process of activation and differentiation to effectors, T cells undergo metabolic reprogramming and shift from anabolic to catabolic mode 30. Autophagy has emerged as a crucial regulator of T cell catabolic activity 31. Deletion of *Atg7*, *Atg5* or *Atg3* impairs CD4^+^ and CD8^+^ T cell proliferation and function in knockout mice 28, 32, 33, whereas deletion of *Atg7* or *Atg5* leads to Treg depletion and greater antitumor response 34. Autophagy also promotes invariant natural killer T (iNKT) cells and Tregs differentiation in the thymus 35. Hence, autophagy regulates the dynamic nature of antitumor immunity and homeostasis.

To determine whether autophagy changes impact tumor progression, most reports were centered on how tumors exploit their intrinsic autophagy competency to survive antitumor immunity in the hostile tumor microenvironment (TME) 36, 37. In contrast, our limited understanding of the effect of host autophagy on the function and integrity of immune mediators that promote tumor progression versus mediators that promote tumor rejection is mainly derived from *in vitro* immune cell culture systems and therefore limited. In other words, mechanisms by which host autophagy stimulates or limits the immune system for recognizing and fighting tumor cells in tumor rejection remain unclear 38, 39. Here we utilized an *in vivo* model in which both autophagy-attenuated *Atg5^flox/flox^* and autophagy-competent *Atg5^+/+^* mice were orthotopically allografted with syngeneic MST cells 40 to examine the role of attenuated host autophagy in regulating antitumor immune response within TME. For the first time, we present evidence that autophagy was associated with a reduction in intratumor glutamine level and suppressed cytotoxic T lymphocyte activity, favoring MST progression. Lastly, dietary glutamine supplementation retarded tumor growth and enhanced host antitumor immunity. Our findings provide a rationale for dietary glutamine supplementation as a therapeutic strategy to exploit the metabolic vulnerability of T cells against MST.

## Material and Methods

### Mice breeding

All animal protocols were in accordance with the guideline of Institutional Animal Care and Use Committee at City of Hope (IACUC 06038). Mice were housed in a specific pathogen-free room with a 12-h light/dark cycles and were fed an autoclaved chow diet and water *ad libitum*. *LGL-KRAS^G12V^* mice, Ela-CreERT mice, and *Atg5^flox/flox^* mice 13 were crossed to derive *Ela-CreERT;LGL-KRAS^G12V^;Atg5^flox/flox^* (*KRAS^G12V^;Atg5^flox/flox^*), and *Ela-CreERT;LGL-KRAS^G12V^; Atg5^+/+^* (*KRAS^G12V^; Atg5^+/+^*) mice, as we described previously 40. Genotyping was conducted as described previously 41, 42. Adult male mice, 8-10 weeks of age, were used in all experiments.

### Diet

All mice were kept on normal chow until start of the experiments. Diets used in this study are based on the open standard diet with 16 %kcal fat with crystalline amino acids from Research Diets Inc. (New Brunswick, NJ, USA). The Control diet (A11112201) contained all essential amino acids and nonessential amino acids as specified by Research Diets. Glutamine-supplemented diet contained all amino acids equal to the control diet with the addition of 200 g of glutamine by Ishak Gabra 43. Corn starch content was adjusted to achieve the isocaloric intake. Mice were fed with respective diets for 28 days. Glutamine concentration was determined in the collected serum and harvested submandibular glands (SMGs), respectively.

### Tumor digests and submandibular glands tumor cells isolation

Tumors were cut into small pieces and digested into single cell suspension as previously described 40. The tumors were minced and digested up to 60 min at 37 ℃ in digestion medium containing collagenase (1 mg/ml; MilliporeSigma, C6885), hyaluronidase (100 units/ml; MilliporeSigma, H3506), DNase I (50 μg/ml; MilliporeSigma, D4527), bovine serum albumin (1 mg/ml; MilliporeSigma, A2153), HEPES (pH 7.3, 20 mM; Corning, 25-060-CI) in Dulbecco's Modified Eagle's Medium/Ham's F-12 50/50 Mix (Corning, 16-405-CV). The suspension of digested tumor cells was passed through a 100 μm sieve to remove the remaining tissue chunks. The red blood cells were lysed by incubating cell suspensions in 1X red blood cell lysis buffer (155 mM NH_4_Cl, 12 mM NaHCO_3_, 0.1 mM EDTA, pH 7.3) for 3 min on ice.

### Primary tumor cell culture

The primary cells were plated on collagen I-coated dishes (Corning, 354450), and maintained in a medium consisting of Dulbecco's Modified Eagle's Medium (Corning, 10-013-CV) with fetal bovine serum (10%; Thermo Fisher Scientific, 10437028), L-glutamine (5 mM; Thermo Fisher Scientific, A2916801), hydrocortisone (400 ng/ml; MilliporeSigma, H0888), insulin (5 μg/ml; Thermo Fisher Scientific, 12585014), EGF (20 ng/ml; Thermo Fisher Scientific, PHG0311), HEPES (15 mM; Thermo Fisher Scientific, 15630080) and antibiotic-antimycotic (1X; Thermo Fisher Scientific, 15240112). After 1 to 2 week of incubation, colonies of the GFP-negative tumor cells were manually picked and transfer to new cell culture dishes.

### Orthotopic tumor implantation

To distinguish host genotypes from genotypes of inoculated tumor cells, the tumor cells collected from *KRAS^G12V^; Atg5^+/+^* and *KRAS^G12V^; Atg5^flox/flox^* tumor-bearing mice were designated as *KRAS^G12V^;Atg5^+/^*^+^ and *KRAS^G12V^;Atg5^∆/∆^*, respectively. Whereas host genotypes were designated as *Atg5^+/+^* and *Atg5^flox/flox^*. *Atg5^+/+^* and *Atg5^flox/flox^* mice were orthotopically inoculated with 2x10^5^ MST cells (*KRAS^G12V^;Atg5^+/+^* and *KRAS^G12V^;Atg5^∆/∆^*), suspended in DMEM/Matrigel (1:1), in the right (SMGs). Tumor sizes were measured at least three times a week with digital calipers and tumor volume was calculated using the formula Volume (mm^3^) = (W^2^ x L)/2, where W (width) and L (length) correspond to the smaller and larger of two perpendicular axes, respectively. Animals were euthanized post-tumor implantation at either early end-point (Day 14) or late end-point (Day 25) according to humane endpoints as specified by COH IACUC guideline. For high glutamine diet feeding experiments, 5x10^4^ MST cells were implanted in SMGs of mice. Tumor-bearing mice were fed with regular or high glutamine diet for another 21 days. Diets were changed weekly, and the consumption of diets were measured. Tumor-bearing mice were euthanized at 21 days post-tumor implantation.

### LPS treatment

Naïve mice were intraperitoneal injected with 5 mg/kg body weight lipopolysaccharides (LPS; MilliporeSigma, LPS25) in PBS or equal volume of PBS. Six hours following LPS administration, spleens were harvested, and spleen weights measured.

### Tissue preparation and characterization

Tumors were excised and fixed in 10% neutral-buffered formalin (MilliporeSigma, HT501128) for 48 h. Tissue embedding, sectioning, and staining with modified Mayer's hematoxylin (American MasterTech, HXMMHGAL) and eosin Y stain (American MasterTech, STE0157), or H&E stain, were performed in City of Hope Pathology Core as previous described 40.

### Immunohistochemistry and quantification

The immunohistochemistry (IHC) was performed by City of Hope Pathology Core as described previously 40-42. Briefly, formalin fixed paraffin embedded (FFPE) tumor tissue slides were deparaffinized and hydrated through xylenes and graded alcohol solutions. The tissue slides were pressure-cooked in citrate-based unmasking solution for 30 min and washed in phosphate-buffered saline for 5 min, followed by quenching of endogenous peroxidase activity in H_2_O_2_ (0.3%; MilliporeSigma, H1009) for 30 min. The slides were then blocked for 20 min with a mixture of Avidin D solution and diluted normal blocking serum, which was prepared from the species in which the secondary antibody is made. The slides were then incubated with a mixture of primary antibody and biotin solution for 30 min and washed in buffer 3 times. The slides were incubated in the Vector biotinylated secondary antibody for 30 min, washed for 5 min, and then incubated in Vectastain Elite ABC Reagent for 30 min. After being washed for 5 min, the slides were processed with the DAB Substrate Kit. Primary antibodies for IHC include antibody recognizing Ki67 (abcam, ab15580), F4/80 (Bio-Rad, MCA497R), CD11b (Abcam, ab133357), CD4 (Biolegend, 201501), CD8 (Thermo Fisher, 14-0808-82). For quantification, 10x magnification images of 5 nonoverlapping fields of tumors (5 images per mouse) were quantified using Image-Pro Premier 9.02 (Media Cybernetics).

### qRT-PCR

RNA was extracted using Trizol (Invitrogen, 15596026) according to the manufacturer's instructions. The concentration of the isolated RNA and the ratio of absorbance at 260 nm to 280 nm (A260/A280 ratio) were measured with spectrophotometer (Biotek). cDNA was generated using iScript Kit (Bio-Rad, 1708890) and the qRT-PCR reaction utilized the components contained in the iTaq Universal SYBR Green Supermix (Bio-Rad, 1725120). Household gene transcript levels (*Gapdh*) were used for normalization. The 2-ΔΔCt method was used to analyze the relative changes in each target gene expression 44. Sequences of the primers are listed in Table S1.

### Immunoblotting

Whole tissue protein was extracted by Qproteome Mammalian Protein Prep Kit (Qiagen, 37901) according to the manufacture's guidelines. Cell lysates were prepared by directly lysing cells in 1X Laemmli SDS-PAGE buffer with vortexing and heating at 95 °C for 10 min. The protein concentration was measured by Bicinchoninic acid assay. Western blotting was performed by running equal amount of protein on a SDS-PAGE gel and immunoblotted with primary antibodies of interest followed by horseradish peroxidase conjugated secondary antibody following manufacturer's instruction. After chemiluminescent reaction, blots were visualized with a Chemi-Doc Touch Imaging System (Bio-Rad).

### Multicolor flow cytometry

Cell suspensions (splenocytes and SMG tumor cells) were stained in FACS buffer (PBS supplemented with 1% BSA) for 30 min on ice using the following antibodies: CD11b-APC-eFluor 780 (Invitrogen, 47-0112-80), F4/80-eFluor 450 (Invitrogen, 48-4801-80), MHCII-PerCP-eFluor 710 (Invitrogen, 46-5320-80), CD86-PE-Cyanine7 (Invitrogen, A15412), CD206-PE (Invitrogen, 12-2061-82), Ly6b-APC (Novus, NBP2-13077APC), NK1.1-Super Bright 702 (Invitrogen, 67-5941-82), CD49b-APC (Invitrogen, 17-5971-81), CD4-PE-Cyanine7 (Invitrogen, 25-0041-81), and CD8-eFluor 450 (Invitrogen, 48-0081-80), diluted in FACS buffer at 1:100 ratio, whereas CD25-SB600 (Invitrogen, 63-0251-80) diluted in FACS buffer at 1:50 ratio. For intracellular cytokine staining, cells isolated from *Atg5^+/+^* and *Atg5^flox/flox^* mice were treated with 50 ng/ml PMA, 750 ng/ml ionomycin (both from Sigma-Aldrich), and GolgiPlug (BD Biosciences) in complete medium at 37 °C for 4-6 h. Cells were fixed and permeabilized with TF Fixation/Permeabilization solution (Invitrogen) before interferon gamma (IFN-γ)-APC (Invitrogen, 17-7311-81, 1:160 dilution) staining, and Foxp3-PE-Cy7 (Invitrogen, 25-5773-82, 1:80 dilution) staining, respectively. LIVE/DEAD™ Fixable Aqua Dead Cell Stain Kit (Invitrogen, L34957) was used to distinguish live and dead cells. Dead cells and doublets were excluded from all analysis. Multiparameter analysis was performed on Attune NxT Acoustic flow cytometer (Invitrogen) and analyzed with the FCS express 7 software (De Novo Software, Glendale, CA).

### CD4^+^ T cell isolation and* in vitro* iTreg differentiation

Naïve mouse CD4^+^ T cells were isolated from spleens of *Atg5^+/+^* and *Atg5^flox/flox^* mice using Naïve CD4^+^ T Cell Isolation Kit (Miltenyi Biotec, 130-104-453). Cells (5 × 10^5^ cells in one milliliter per well) were seeded in 24-well plate pre-coated with 0.05 mg/ml goat anti-hamster antibody (MP Biomedicals) at 4°C overnight. iTreg induction medium is glutamine-free RPMI 1640 supplemented with 10% fetal bovine serum (FBS, Gibco), 2-mercaptoethanol (50 μM), penicillin (100 U/ml), streptomycin (100 μg/ml, Corning), hamster anti-CD3 (0.25 µg/ml, eBioscience, 145-2C11), hamster anti-CD28 (1 µg/ml, eBioscience, 37.51), TGF-β (3 ng/ml, Peprotech), and mIL-2 (100 U/ml, Biolegend). Fresh media with escalating concentrations of glutamine were replenished every day for 3 days. Cells were then stained with viability dye, CD4, CD25, Foxp3, and followed by flow cytometric analysis.

### Tumor tissue and plasma glutamine quantification

Glutamine extraction and quantification from tumor tissue was modified from method by Pan et al. 45. Approximately 50 mg of fresh tumor tissues were homogenized in ice cold 70% ethanol using TissueLyser II (Qiagen). After spinning down, the pellet was collected and dried using SpeedVac vacuum concentrator. The dried pellet was then resuspended in phosphate buffer (20 mM, pH 7.2, 500 μl) and centrifuged to remove debris. The supernatant (400 μl) was transferred and dried with speed vacuum. Subsequently, the dried pellet was dissolved in 550 μl D_2_O containing 0.01 mg/ml Sodium 2,2-Dimethyl-2-Silapentane-5-Sulfonate (DSS; Cambridge Isotope). The samples were then vortexed and centrifuged at 16000 rpm for 12 min. 500 μl solution was transferred to NMR tube. The NMR experiments were carried out at 25°C on a Bruker 700 MHz Avance spectrometer equipped with cryoprobe. To suppress residual macromolecule signals, a Carr-Purcell- Meiboom-Gill (CPMG) sequence with Periodic Refocusing Of J Evolution by Coherence Transfer (PROJECT) method 46 and pre-saturation was used to acquire the 1D ^1^H data. The spectrum width is 13.4 ppm, the recycle delay, acquisition time are 1.5 and 3.5 seconds, respectively. The CPMG duration is 250 ms with 52 echoes and 1.2 ms delay between pulses in the CPMG echo. A control sample with known concentration of glutamine and glutamate and internal reference DSS was prepared to determine the CPMG effect on the peak intensity of glutamine and glutamate. The glutamine and glutamate concentration from tissue extractions was first determined using Chenomx software, and then adjusted by taking account of CPMG effect. Glutamine concentration of the plasma samples was measured by EnzyChrom Glutamine Assay Kit (BioAssay Systems, EGLN-100) following the protocol of the manufacturer. Mouse plasma was collected and diluted two-fold in PBS. To 30 μl of diluted plasma, 15 μl of inactivation solution (0.6 N HCl) was added, mixed, and incubated for 5-10 min at room temperature. Then 15 μl of Tris solution (600 mM, pH 8.5) was added and proceeded with the EnzyChrom Assay.

### αKG extraction and measurement

Alpha-ketoglutarate (αKG) extraction from tumor tissue was as described above. α-ketoglutarate was quantified using αKG Assay Kit (Abcam, ab83431) following the manufacture's protocol.

### Immune depletion of CD8^+^ and CD25^+^ cells

For the depletion of CD8^+^ cells, *Atg5^+/+^* and *Atg5^flox/flox^* mice were treated with an anti-CD8a (0.2 mg/mouse; Bio X Cell, clone 2.43) depleting antibody or a control IgG (0.2 mg/mouse; Bio X Cell, BP0090) intraperitoneally (*i.p.*) in 0.1 ml PBS at Days -1, +1, +7 and +15 pre- and post-tumor cell implantation. For the depletion of CD25^+^ cells, *Atg5^+/+^* mice were treated with anti-CD25 depleting antibody (0.25 mg/mouse; Bio X Cell, clone PC-61.5.3) or a control IgG (0.25 mg/mouse; Sigma, I4131) intraperitoneally at Days -1, +3, +10 and +17 relative to tumor cell implantation. MST cells (5x10^4^) were orthotopically implanted in the SMGs of mice at Day 0.

### Statistical analysis

Data are represented as mean and standard error of mean (Mean ± SEM). Statistical analyses were performed using GraphPad Prism 7.0 software (GraphPad Prism Software Inc., San Diego, CA). For all experiments, at least three independent biological replicates of each condition were analyzed. The comparison between two means was analyzed by a two-tailed unpaired Student's *t*-test or Welch's *t* test, one tailed, unpaired. The *p*-value, which is < 0.05, was considered statistically significant. Center value is defined as mean value, and standard error of the mean (s.e.m.) is used to calculate and plot error bars from raw data.

## Results

### Attenuated autophagy is sufficient for the suppression of MSTs

**Figure 1:**
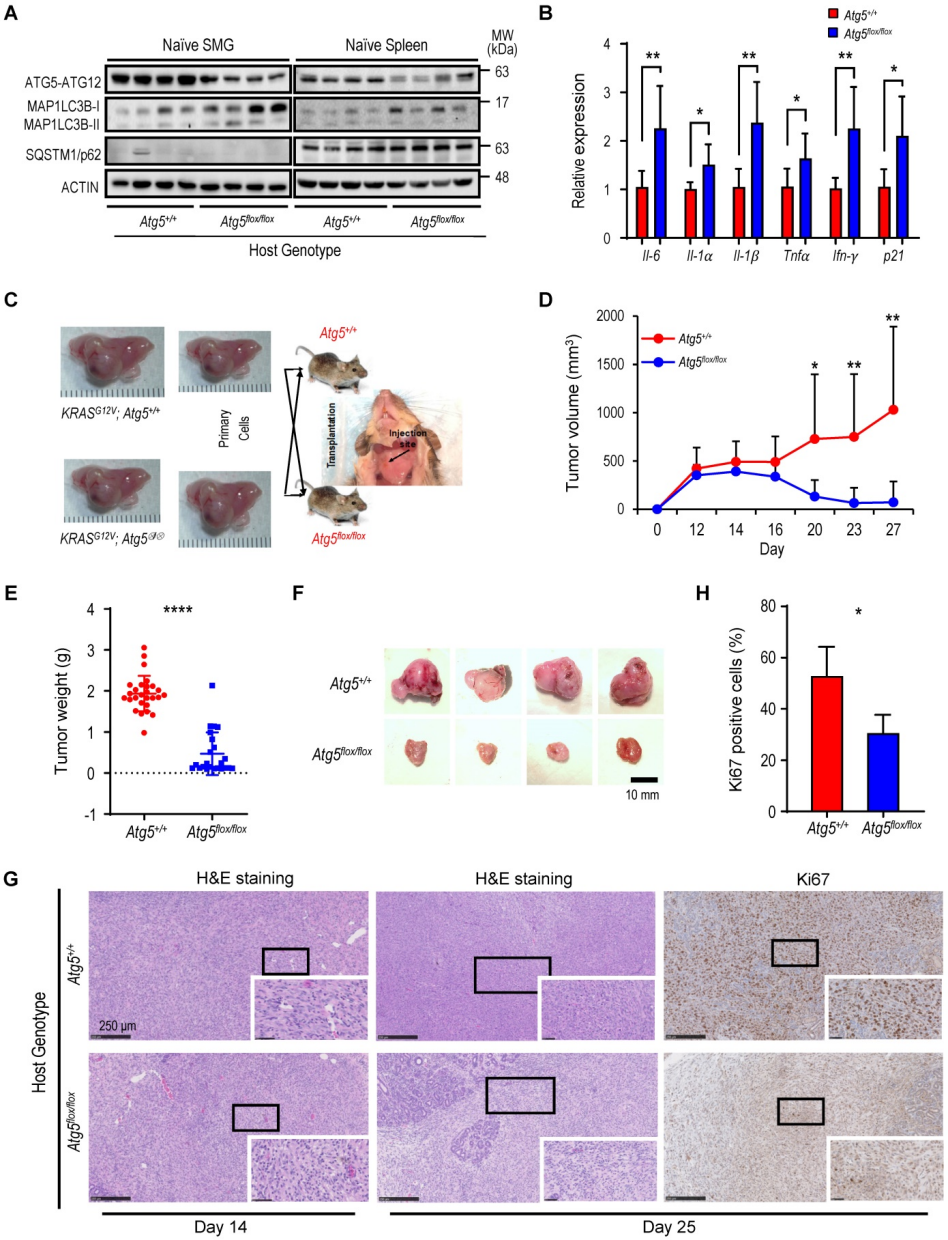
** Attenuated autophagy is essential for the suppression of malignant salivary tumors.** (**A**) Autophagy activity was verified in SMGs and spleens from *Atg5^flox/flox^* mice by determining the expression of ATG5 and decreased ratio of LC3-II/I. A representative Western blot analysis of ATG5 and basal LC3 in SMGs and spleens from *Atg5^+/+^* and *Atg5^flox/flox^* mice following salivary tumor cell inoculation. (**B**) Quantitative RT-PCR analyses show basal expression of selected proinflammatory cytokine genes in SMGs from naïve *Atg5^+/+^* (n = 3) and *Atg5^flox/flox^* (n = 5) mice. (**C**) Schematic diagram of orthotopic allograft of salivary tumor cells in right SMGs. Host genotypes are designated as *Atg5^+/+^* and *Atg5^flox/flox^*, while the injected tumor cell genotypes are designated as *KRAS^G12V^;Atg5^+/+^* and *KRAS^G12V^;Atg5^∆/∆^*. (**D**, **E**, **F**) Compromised host autophagy reduces orthotopically implanted salivary tumor expansion. (**D**) Tumor volumes were recorded at 2-day intervals. A representative tumor growth curve is shown. The limitation in tumor growth in host recipients with ATG5 deficiency was observed starting from Day 16 following salivary tumor cell inoculation. (**E, F**) Tumor-bearing SMG weights were measured, and images were taken at Day 25 post-tumor cell inoculation or at humane endpoints. *Atg5^+/+^* and *Atg5^flox/flox^* mice were injected with 2 x 10^5^ primary tumor cells (*KRAS^G12V^;Atg5^+/+^* and *KRAS^G12V^;Atg5^∆/∆^*) in right submandibular glands of *Atg5^+/+^* (n = 18) and *Atg5^flox/flox^* (n = 15) mice (**E**). Representative images of salivary tumors harvested from of *Atg5^+/+^* and *Atg5^flox/flox^* mice (**F**). (**G**) Hematoxylin and eosin (H&E) staining and Ki-67 immunohistochemical staining of SMG tumors. At Day 14 and Day 25 post-implantation, SMGs tissue samples from *Atg5^+/+^* and *Atg5^flox/flox^* mice were collected, processed, and stained with H&E and an anti-Ki-67 antibody for IHC. (**H**) Quantification of Ki-67^+^ cells is as shown (*Atg5^+/+^*: n = 6; *Atg5^flox/flox^*: n = 4). Five random low-power fields were quantified from each mouse. Scale bar, 250 μm and 50 μm (enlarged view); respectively. Data are shown as mean ± SD; *: *p* < 0.05; **: *p* < 0.01; ****: *p* < 0.0001; Student's *t*-test, 2-tailed, unpaired.

To investigate the role of autophagy in regulating MST progression at various stages, we developed an inducible *KRAS^G12V^;Atg5^flox/flox^* mouse model with an ability for conditional activation of oncogenic KRAS^G12V^ and disruption of the essential autophagy protein ATG5 in submandibular glands (SMGs) 40-42. We showed that ATG5-knockout tumors grow more slowly during late tumorigenesis, despite a faster onset 40. MST cells were isolated from both* KRAS^G12V^;Atg5^+/+^* and *KRAS^G12V^;Atg5^∆/∆^* tumors, respectively for biochemical analyses (**Figure S1A**). In *KRAS^G12V^;Atg5^∆/∆^* tumor cells, the *Atg5* expression was ablated and the conversion of microtubule-associated protein 1A/1B-light chain 3 (LC3)-I to the lipidated form of LC3B-II was lower than *KRAS^G12V^;Atg5^+/+^* MST cells (**Figure S1B**), supporting the deletion of ATG5. Next, to investigate the effect of host *Atg5* genotype on autophagy competency, we compared autophagy parameters between SMGs and spleens from naïve *Atg5^+/+^* and *Atg5^flox/flox^* mice. As shown in **Figure 1A**, a reduction, but not depletion, of ATG5-ATG12 and an accumulation of LC3-I confirmed the attenuated autophagy in SMGs and spleens from naïve *Atg5^flox/flox^* mice. Autophagy plays a crucial role in modulating immune system homeostasis 21-25. Several key autophagy components participate in the immune and inflammatory processes; more specifically, the ATG5-ATG12 conjugate is associated with innate antiviral immune responses 47, 48. Consistent with this, the basal expression of proinflammatory cytokine genes, *Il-6*, *Il-1α*, *Il-1β*, *Tnfα*, *Ifn*-γ and *p21,* was significantly higher in SMGs of naïve *Atg5^flox/flox^* mice when compared to SMGs of naïve *Atg5^+/+^* mice (**Figure 1B**).

Next, we hypothesized that an attenuated host autophagy is a barrier for tumor progression in SMGs. To test this possibility, we investigated host tumor microenvironment following the inoculation of *KRAS^G12V^;Atg5^+/+^* and *KRAS^G12V^;Atg5^∆/∆^* tumor cells, respectively, into different genotypic recipient mice (*Atg5^+/+^* and *Atg5^flox/flox^*; **Figure 1C**). There was no noticeable difference in tumor expansion between *KRAS^G12V^;Atg5^+/+^* and *KRAS^G12V^;Atg5^∆/∆^* tumor cells in host with the same genotype (lane 1 vs lane 2, lane 3 vs lane 4; **Figure S1C**). We therefore chose to use *KRAS^G12V^;Atg5^∆/∆^* tumor cells in the subsequent studies for consistency. Tumors derived from the inoculated MST cells exhibited similar histopathological features as the endogenous tumors (**Figure S1D**). In contrast, a significant reduction in tumor growth, starting from Day 16 following tumor cell inoculation was observed in* Atg5^flox/flox^* recipient mice, compared to *Atg5^+/+^* recipient mice (**Figure 1D**). Accordingly, SMG tumor weights from *Atg5^flox/flox^* recipient mice were significantly lower than those from *Atg5^+/+^* recipient mice at the later time-point when tumors were harvested (**Figure 1E-F, S1C**). H&E staining shows that tumors from *Atg5^flox/flox^* recipient mice often exhibited reduced progression as normal salivary tissues were abundantly detected within SMGs, whereas the SMGs from* Atg5^+/+^* recipient mice had fewer regions displaying normal salivary tissues at Day 14 and Day 25 post-MST cell inoculation (**Figure 1G**). Consistently, a decrease in the proliferation marker, Ki-67, was observed in SMGs from tumor-bearing *Atg5^flox/flox^* mice compared to those SMG from tumor-bearing *Atg5^+/+^* mice (**Figure 1G-H**). Of note, tumor growth prior to Day 16 was indistinguishable between two host genotypes (**Figure 1D**). However, tumor volume between recipient hosts consistently diverged after Day 16. At this point, continued growth was noted only in tumor-bearing *Atg5^+/+^* mice. In contrast, tumor regression was seen in tumor-bearing *Atg5^flox/flox^* mice. These findings underscore the importance of antitumor immune response in a host autophagy-specific manner. Subsequent studies were focused on analyzing the TME residents in tumor-bearing *Atg5^+/+^* mice and *Atg5^flox/flox^* mice on Day 14 and on Day 25, respectively.

### Attenuated autophagy suppresses macrophages expansion within TME

Next, we hypothesized that attenuated autophagy promotes antitumor immunity by affecting the infiltrated cell populations in SMGs. To test this hypothesis, we sought to examine whether autophagy regulates TME residents in our orthotopic syngeneic mouse MST model. Based on our observations of a marked infiltration of inflammatory cells, including macrophages and leukocytes in inducible MSTs 41 and elevated proinflammatory cytokines in naïve *Atg5^flox/flox^* mice (**Figure 1B**), we first analyzed and compared leukocytes between tumor-bearing *Atg5^+/+^* and *Atg5^flox/flox^* mice. Flow cytometry analyses of CD11b^+^CD49b^+^NK1.1^+^ NK cells (**Figure S2A**) and CD11b^+^F4/80^-^Ly6B^+^ neutrophils (**Figure S2B**) showed that the frequencies of NK cells and neutrophils within the harvested tumor-harboring SMGs at Day 14 post-tumor cell implantation were not host genotype-dependent (**Figure S2C-D**). At Day 25, NK cell frequency remained the same between different hosts (**Figure S2E**). However, there was a significant decrease in neutrophils in spleens, a major secondary organ in the immune system, but not SMGs of tumor-bearing *Atg5^flox/flox^* mice at Day 25 (**Figure S2F**).

**Figure 2:**
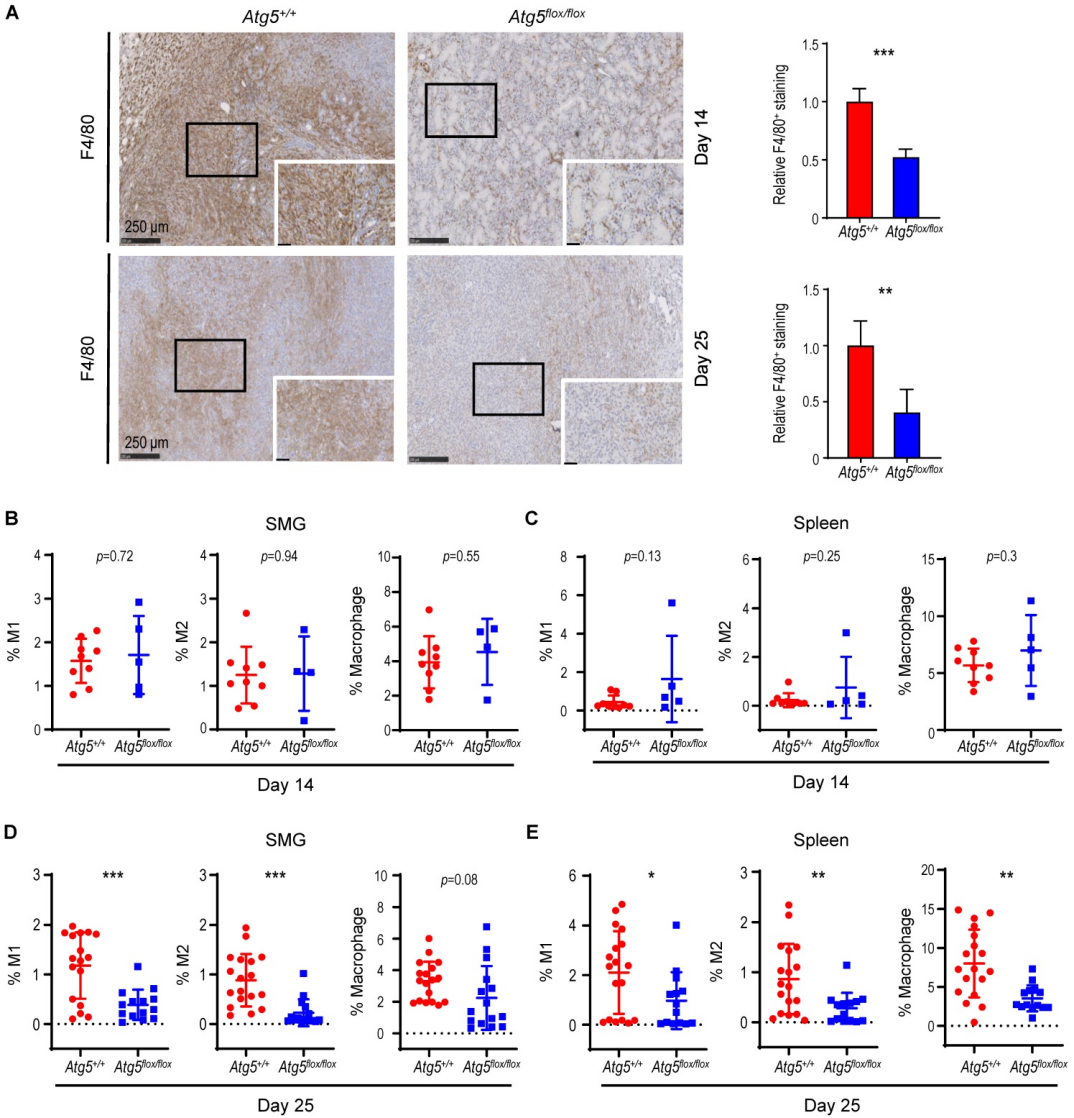
** A decrease in M1 and M2 macrophages within TME in tumor-bearing *Atg5^flox/flox^* mice.** (**A**) Representative immunohistochemical staining of pan-macrophage marker F4/80 were performed on Day 14 (*upper left two panels*) and Day 25 (*lower left two panels*) in SMG tumors from *Atg5^+/+^* and *Atg5^flox/flox^* mice. Scale bar, 250 μm and 50 μm (enlarged view); respectively. Quantification of F4/80 in Day 14 (*upper right panel*) and Day 25 (*lower right panel*) SMG tumors is as shown. Five random low-power fields were quantified from each mouse. (Day 14, *Atg5^+/+^*: n=4 and *Atg5^flox/flox^*: n = 4; Day 25, *Atg5^+/+^*: n = 6 and *Atg5^flox/flox^*: n = 3.) (**B**, **C**) Flow cytometry analyses show the percentage of M1 macrophages (CD11b^+^F4/80^+^MHCII^+^CD86^+^, *left panel*), M2 macrophages (CD11b^+^F4/80^+^MHCII^-^CD206^+^, *middle panel*) and macrophages (CD11b^+^F4/80^+^, *right panel*) in alive SMG cells (**B**) and splenocytes (**C**) of tumor-bearing mice at Day 14 post-implantation. (**D**, **E**) Flow cytometry analyses show the percentage of M1 macrophages (CD11b^+^F4/80^+^MHCII^+^CD86^+^, *left panel*), M2 macrophages (CD11b^+^F4/80^+^MHCII^-^CD206^+^, *middle panel*) and macrophages (CD11b^+^F4/80^+^, *right panel*) in alive SMG cells (**D**) and splenocytes (**E**) of tumor-bearing mice at Day 25 post-inoculation. *Atg5^+/+^*: n = 18 and *Atg5^flox/flox^*: n = 15. Data are shown as mean ± SD; *: *p* < 0.05; **: *p* < 0.01; ***: *p* < 0.001; Student's *t*-test, 2-tailed, unpaired.

We next characterized tumor-associated macrophages (TAMs). Circulating monocytes give rise to mature macrophages that are recruited into the TME and differentiate *in situ* into TAMs upon activation 49. TAMs are further classified into classically activated or pro-inflammatory M1 and alternatively activated or anti-inflammatory M2 macrophages 50. We evaluated the correlation between the autophagy capacity and the abundance of macrophage F4/80 marker in SMGs of *Atg5^+/+^* and *Atg5^flox/flox^* recipient mice using immunohistochemistry (IHC). As shown in **Figure 2A**, a decrease in infiltrating F4/80 counts in SMGs of tumor-bearing *Atg5^flox/flox^* mice at both Day 14 and Day 25 was noted. Flow cytometry analyses further revealed that the percentages of CD11b^+^F4/80^+^MHCII^+^CD86^+^ M1 and CD11b^+^F4/80^+^MHCII^-^CD206^+^ M2 macrophages in SMGs and spleens (controls) at Day 14 were not host-dependent (**Figure 2B-C**). Given that tumor burden at Day 25 was higher in SMGs of *Atg5^+/+^* mice than *Atg5^flox/flox^* counterparts (**Figure 1D**), *Atg5^+/+^* SMGs had increased M1 and M2 macrophage infiltrate compared to *Atg5^flox/flox^* SMGs at Day 25 (**Figure 2D**). Further, M1 and M2 populations were significantly higher in spleens of tumor-bearing *Atg5^+/+^* and *Atg5^flox/flox^* mice at Day 25 (**Figure 2E**). Spleen-derived macrophages were readily polarized into M1 and M2 states, presumably via the tumor-spleen signaling interaction of IFN-γ or other cytokines 51, 52. Together, our data suggests that autophagy promotes the expansion of both M1 and M2 TAMs in SMGs of tumor-bearing *Atg5^+/+^* recipient mice at the later stage post-tumor cell inoculation.

### Attenuated autophagy promotes tumor-infiltrating cytotoxic CD8^+^ T cells and IFN-γ production

**Figure 3:**
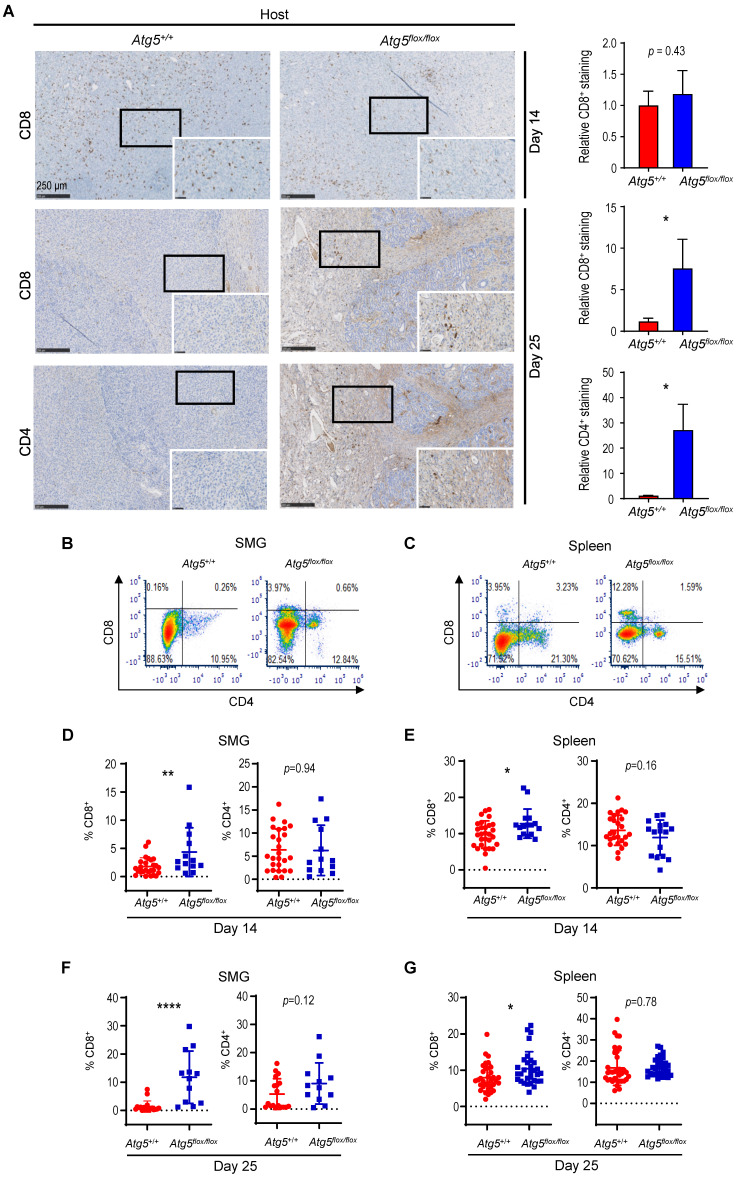
** An enhancement in CD8^+^ T cells within TME in tumor-bearing *Atg5^flox/flox^* mice.** (**A**) Representative immunohistochemistry analyses of CD8 on Day 14 SMG tumors (*upper left panels*), and CD8 (*middle left panels*) and CD4 (*lower left panels*) on Day 25 SMG tumors from *Atg5^+/+^* and *Atg5^flox/flox^* mice. Scale bar, 250 μm and 50 μm (enlarged view); respectively. Quantification of CD8^+^ signals in Day 14 SMG tumors (*upper right panel*) and quantification of CD8^+^ signals (*middle right panel*) and CD4^+^ signals (*lower right panel*) in SMG tumors from Day 25 *Atg5^+/+^* and *Atg5^flox/flox^* mice. Five random low-power fields were quantified from each mouse. *Atg5^+/+^*: n ≥ 4 and *Atg5^flox/flox^*: n ≥ 3. (**B**, **C**) Representative flow cytometry showing CD8^+^ T cells and CD4^+^T cells isolated from alive SMG cells (**B**), and splenocytes (**C**) of tumor-bearing *Atg5^+/+^* and *Atg5^flox/flox^* mice. (**D**-**G**) Flow cytometry analyses showing the percentage of CD8^+^ T cells and CD4^+^ T cells in alive SMG tumor cells, and splenocytes from Day 14 (**D**, **E**) and Day 25 (**F**, **G**) tumor-bearing *Atg5^+/+^* (*red*) and *Atg5^flox/flox^* (*blue*) mice (*Atg5^+/+^* and *Atg5^flox/flox^*: n ≥ 12). Data are shown as mean ± SD; *: *p* < 0.05; **: *p* < 0.01; ****: *p* < 0.0001; Student's *t*-test, 2-tailed, unpaired.

Cancer immune evasion is a major stumbling block for antitumor immunity. We further elucidated the role of autophagy in regulating the infiltration of cytotoxic T lymphocytes into tumors. To achieve this goal, we first focused on CD4^+^ helper and CD8^+^ cytotoxic T lymphocytes. At Day 14 post-tumor cell implantation, there was no significant difference in the frequency of infiltrating CD8^+^ T cells between SMGs of tumor-bearing *Atg5^+/+^* and *Atg5^flox/flox^* mice by IHC (**Figure 3A**). However, a clear increase in the percentage of infiltrating CD4^+^ and CD8^+^ T cells was detected in SMGs of tumor-bearing *Atg5^flox/flox^* mice at Day 25 by IHC (**Figure 3A**). Consistently, flow cytometry (**Figure 3B-C**) confirmed a higher percentage of CD8^+^ T cells in SMGs (**Figure 3D**,** F**, *left panels*) and spleens (**Figure 3E**,** G**, *left panels*), respectively, in tumor-bearing *Atg5^flox/flox^* mice at both Day 14 (**Figure 3D-E**) and Day 25 (**Figure 3F-G**). Conversely, the host autophagy capacity did not affect percentages of CD4^+^ T cells in SMGs and spleens from tumor-bearing mice at Day 14 (**Figure 3D-E**, *right panels*) and Day 25 (**Figure 3F-G**, *right panels*). We conclude that the increased infiltrating CD8^+^ cytotoxic T cells play a key role in the observed antitumor phenotypes in tumor-bearing *Atg5^flox/flox^* mice.

**Figure 4:**
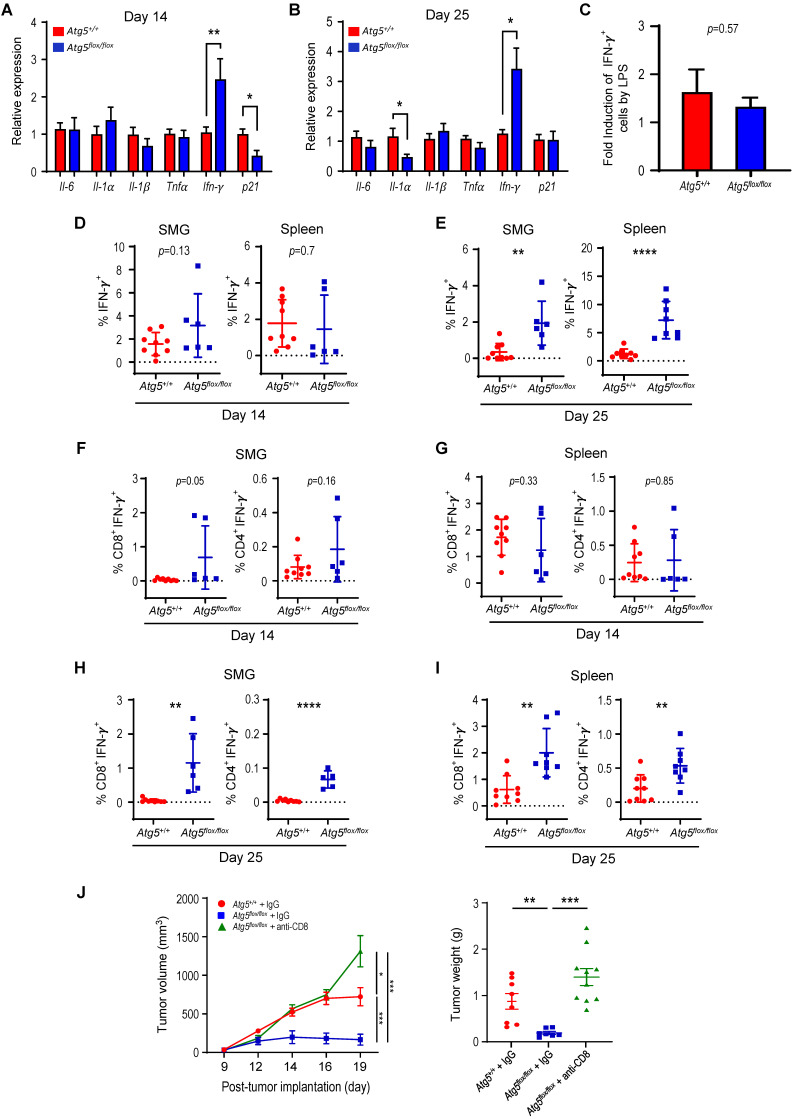
** Attenuated autophagy promotes IFN-γ-producing CD8^+^ and CD4^+^ T cells in SMGs and spleens of tumor-bearing mice.** (**A**) Relative expression of proinflammatory cytokine-related genes in SMGs from tumor-bearing *Atg5^+/+^* and *Atg5^flox/flox^* mice, Day 14 (**A**) and Day 25 (**B**) post-implantation (n = 3). (**C**) Fold induction of IFN-γ-producing cells in spleens of *Atg5^+/+^* and *Atg5^flox/flox^* mice following LPS stimulation (5 mg/kg) for 6 h compared with that from PBS control mice (n = 3). (**D**) The percentage of IFN-γ-producing cells in single cell suspensions of the alive SMG cells (*left panel*) and splenocytes (*right panel*), from Day 14 tumor-bearing *Atg5^+/+^* (*red*) and *Atg5^flox/flox^* (*blue*) mice, following PMA/ionomycin/Golgiplug stimulation for 4 h. (**E**) The percentage of IFN-γ-producing cells in single cell suspensions of the alive SMG resident cells (*left panel*) and splenocytes (*right panel*), from Day 25 tumor-bearing *Atg5^+/+^* (*red*) and *Atg5^flox/flox^* (*blue*) mice, following PMA/ionomycin/Golgiplug stimulation for 4 h. (**F**-**I**) Flow cytometry analyses showing the percentage of IFN-γ^+^ T cells, CD8^+^IFN-γ^+^ cells and CD4^+^IFN-γ^+^, in single cell suspensions of the alive SMG cells and splenocytes from Day 14 (**F**, **G**) and Day 25 (**H, I**) tumor-bearing *Atg5^+/+^* (*red*) and *Atg5^flox/flox^* (*blue*) mice (*Atg5^+/+^* and *Atg5^flox/flox^*: n ≥ 5). Data are shown as mean ± SD; *: *p* < 0.05; **: *p* < 0.01; ****: *p* < 0.0001; Student's *t*-test, 2-tailed, unpaired. (**J**) CD8 ablation reverses tumor rejection in *Atg5^flox/flox^* mice.* Atg5^flox/flox^* and *Atg5^+/+^* mice were inoculated with MST cells and received either an anti-CD8 or IgG antibody (0.2 mg/mouse) at indicated time as shown in **Fig. S5A**. Tumor growth was monitored with a caliper (*left panel*). The average tumor weight is shown on the *right panel* at Day 20 post-tumor implantation. Data are presented in bar graph shown as Mean ± SEM. *p* value was calculated by Welch's *t* test, one tailed, unpaired; *Atg5^+/+^* + IgG: n = 8; *Atg5^flox/flox^* + IgG: n = 7; *Atg5^flox/flox^* + anti-CD8: n = 10; *: *p* < 0.05; **: *p* < 0.01; ***: *p* < 0.001; *n.s.*, not significant.

IFN-γ is key to cellular immune responses and is secreted predominantly by activated lymphocytes, such as CD4^+^ helper and CD8^+^ cytotoxic T cells 53. We next assessed expression of proinflammatory cytokine-related genes in SMGs from tumor-bearing recipient mice by qRT-PCR and found increased IFN-γ expression in *Atg5^flox/flox^* recipient mice at both Day 14 and Day 25 (**Figure 4A-B**). However, the IFN-γ production by splenocytes from the naïve *Atg5^+/+^* and *Atg5^flox/flox^* mice challenged with lipopolysaccharide (LPS), to stimulate the release of proinflammatory cytokines, were comparable (**Figure 4C**). We conclude that different host autophagy capacity did not alter IFN-γ production from splenocytes upon LPS challenge. Next, we treated isolated SMG resident cells and splenocytes with a leukocyte activation cocktail containing PMA/ionomycin/Golgiplug 54 to promote intracellular cytokines accumulations, and assessed IFN-γ-production by flow cytometry (**Figure S3A-B**). Notably, there was a significant increase in the frequency of IFN-γ-producing cells in SMGs and spleens of tumor-bearing *Atg5^flox/flox^* mice at Day 25 (**Figure 4E**), but not at Day 14 (**Figure 4D**). Further, no changes in CD8^+^IFN-γ^+^ and CD4^+^IFN-γ^+^ populations in SMGs (**Figure 4F**), and spleens (**Figure 4G**) from *Atg5^+/+^* and *Atg5^flox/flox^* recipient mice were observed at Day 14. In contrast, *Atg5^flox/flox^* recipient mice had higher frequencies of CD8^+^IFN-γ^+^ cells in SMGs and spleens, respectively, at Day 25 (**Figure 4H-I**, *left panels*). CD4^+^IFN-γ^+^ cells, albeit with reduced frequencies, were also higher in SMGs and spleens of *Atg5^flox/flox^* recipient mice (**Figure 4H-I**, *right panels*). It is conceivable that the increased IFN-γ production by cytotoxic CD8^+^IFN-γ^+^ cells improved the antitumor responses in SMGs of *Atg5^flox/flox^* recipient mice.

It is plausible that an increased CD8^+^ population could result in an improved antitumor immunity in *Atg5^flox/flox^* mice. To test this possibility, we depleted CD8^+^ cells by treating tumor-bearing *Atg5^flox/flox^* mice with an anti-CD8 neutralizing antibody (**Figure S5A**). We found that the tumor volume and tumor weight in tumor-bearing *Atg5^flox/flox^* mice treated with an anti-CD8 antibody significantly increased, comparable to those in IgG-treated tumor-bearing *Atg5^+/+^* mice, over IgG-treated tumor-bearing *Atg5^flox/flox^* mice (**Figure 4J**). The depletion efficiency was monitored in the spleens and tumor-bearing SMG tissues by flow cytometry (**Figure S5B**). Consistent with prior results (**Figure 3D-G**), percentage of CD8^+^ cells is higher in IgG-treated *Atg5^flox/flox^* mice than the same strain of mice treated with anti-CD8 antibody. Administration of anti-CD8 antibody significantly reduced CD8^+^ cells in spleens and tumor-bearing SMG tissues in *Atg5^flox/flox^* mice, while the percentage of CD4^+^ cells remained unaffected (**Figure S5B**). To further delineate the role of CD4^+^/CD8^+^ cells in tumor rejection in *Atg5^flox/flox^* mice, the PC-61.5.3 antibody was used to deplete CD25^+^ cells in tumor-bearing *Atg5^+/+^* mice (**Figure S5C**). However, we observed a very modest, if any, effect on tumor burden in *Atg5^+/+^* mice by this anti-CD25 monoclonal antibody (**Figure S5D-E**). These findings are consistent with the model that CD8^+^ T cells are the major cell type to mediate antitumor activities in tumor-bearing *Atg5^flox/flox^* mice.

### Glutamine-dependent regulation of Treg cells in SMGs and spleens

**Figure 5:**
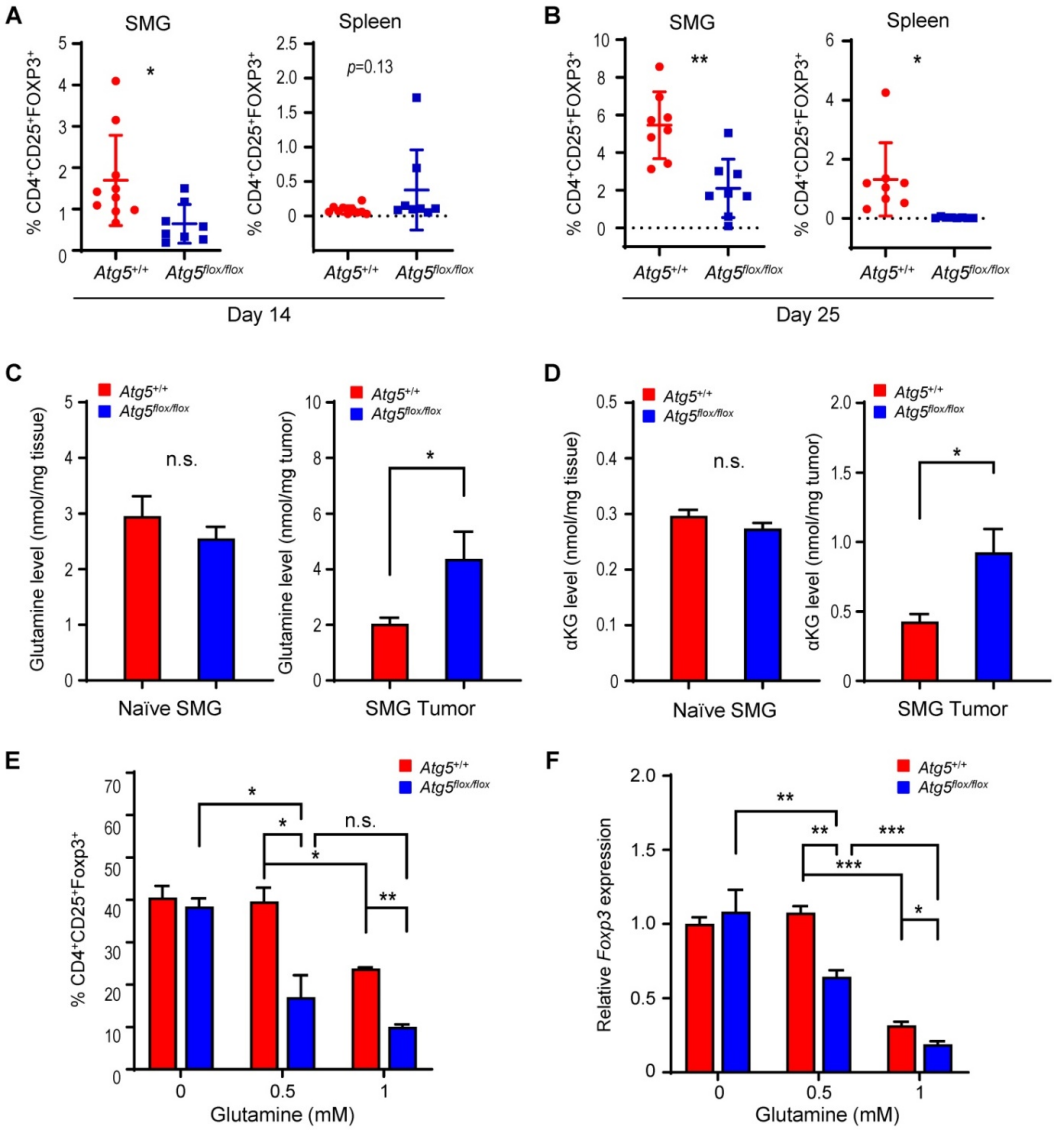
** Attenuation of Treg population in SMGs and spleens of *Atg5^flox/flox^* mice is associated with the glutamine concentration in SMG tumor microenvironment.** (**A, B**) Flow cytometry analyses showing CD4^+^CD25^+^Foxp3^+^ Tregs in single cell suspensions of the SMG cells (*left panels*) and splenocytes (*right panels*) of tumor-bearing mice, Day 14 (**A**) and Day 25 (**B**) after tumor implantation (n ≥ 8). (**C, D**) Levels of glutamine (**C**) and α-ketoglutarate (αKG; **D**) in naïve SMGs (*left panels*) and Day 14 SMG tumors (*right panels*). Glutamine and αKG concentrations in SMG tumors and SMGs from naïve mice were respectively determined (n ≥ 5). (**E**) The percentage of CD4^+^CD25^+^Foxp3^+^ Tregs, a subset of CD4^+^ T cells, is negatively correlated with glutamine concentration in Treg polarization medium. Naïve mouse CD4^+^ T cells were isolated from mouse spleen and cultured for 3 days in Treg polarization medium with indicated glutamine concentrations. The percentages of Foxp3^+^ cells of total CD4^+^CD25^+^ cells are indicated in the bar graph (n = 3). (**F**) Levels of *Foxp3* mRNA expression in the induced Tregs after cultured in Treg polarization medium with indicated glutamine concentrations for the indicated genotypes. Naïve mouse CD4^+^ T cells were isolated from spleens of *Atg5^+/+^* (*red*) and *Atg5^flox/flox^* (*blue*) mice and cultured 3 days in Treg polarization medium. Expression of Foxp3 mRNA isolated from the differentiated cells was analyzed by qRT-PCR (n = 3). Data are presented in bar graph shown as Mean ± SEM. *p* value was calculated by *t* test (unpaired, two tailed). *: *p* < 0.05; **: *p* < 0.01; ***: *p* < 0.001; *n.s.*, not significant.

Subsets of T cells within TME play distinct roles in mediating antitumor immunity 55. Upon tumor antigen stimulation, naïve T cells are activated and differentiate into two broad classes of CD4^+^ or CD8^+^ T cells that have distinct effector mechanisms 12. One CD4^+^ T cell subset, Tregs, dampens the antitumor immune response 18. We next examined the effect of host autophagy capacity on Treg population within TME of *Atg5^+/+^* and* Atg5^flox/flox^* recipient mice. A lower count of CD4^+^CD25^+^Foxp3^+^ Tregs in SMGs at Day 14 was detected in tumor-bearing *Atg5^flox/flox^* mice (**Figure 5A**, *left panel*), while the Treg counts in spleens from mice with different host autophagy capacity were indistinguishable (**Figure 5A**, *right panel*). Notably, **Figure 5B** showed a significant decrease in Tregs in SMGs and spleens from tumor-bearing *Atg5^flox/flox^* mice at Day 25. During T cell activation, glutamine metabolism increases to meet rapid growth requirement 56. We have previously shown that autophagy deficiency contributes to reduced intracellular concentration of most amino acids except glutamine, the level of which increased in *KRAS^G12V^;Atg5^∆/∆^* tumor cells 40. Given that autophagy inhibition promotes glutamine uptake 57, we examined whether glutamine level regulates T cell differentiation into specific subtypes in SMGs of *Atg5^+/+^* and* Atg5^flox/flox^* recipient mice. Notably, both glutamine (**Figure 5C**, *right panel*) and its metabolite α-ketoglutarate (αKG) (**Figure 5D**, *right panel*) levels were higher in SMGs from tumor-bearing *Atg5^flox/flox^* mice at Day 14 comparing to tumor-bearing *Atg5^+/+^* mice. In contrast, there was no difference in glutamine and αKG detected in SMGs of naïve *Atg5^+/+^* and *Atg5^flox/flox^* mice (**Figure 5C-D**, *left panels*). **Figure 5E** showed that naïve T cells differentiated into Tregs in a reverse glutamine-dependent manner under Treg polarization conditions, suggesting that glutamine shortage would render a higher frequency of CD4^+^CD25^+^Foxp3^+^ Tregs. This finding was consistent with the decreased *Foxp3* expression in Tregs differentiated under increasing glutamine concentrations in polarization medium (**Figure 5F**). Interestingly, there was an increase in the frequency of IFN-γ secreting CD4^+^ T cells derived from naïve T cells of *Atg5^flox/flox^* mice at 24 h and 48 h in glutamine-replenished Treg polarization medium (**Figure S4**). Conceivably, glutamine concentration in SMGs not only regulated Tregs population but also IFN-γ secreting CD4^+^ T cells. Together, **Table 1** summarizes the comparison between tumor growth and TME residents from *Atg5*^+/+^ and *Atg5^flox/flox^* recipient mice at Day 14 and Day 25. Tumors continued to grow in the *Atg5*^+/+^ recipient mice while tumors regressed in *Atg5^flox/flox^* recipient mice after 14 days following cell implantation. A statistically significant decrease in CD4^+^ subpopulation, Tregs, and an increase in CD8^+^ T cells were noted (**Table 1**), supporting the role of attenuated host autophagy in promoting antitumor immune responses in MST-bearing *Atg5^flox/flox^* mice.

### Dietary glutamine supplementation is sufficient to suppress MST

**Figure 6:**
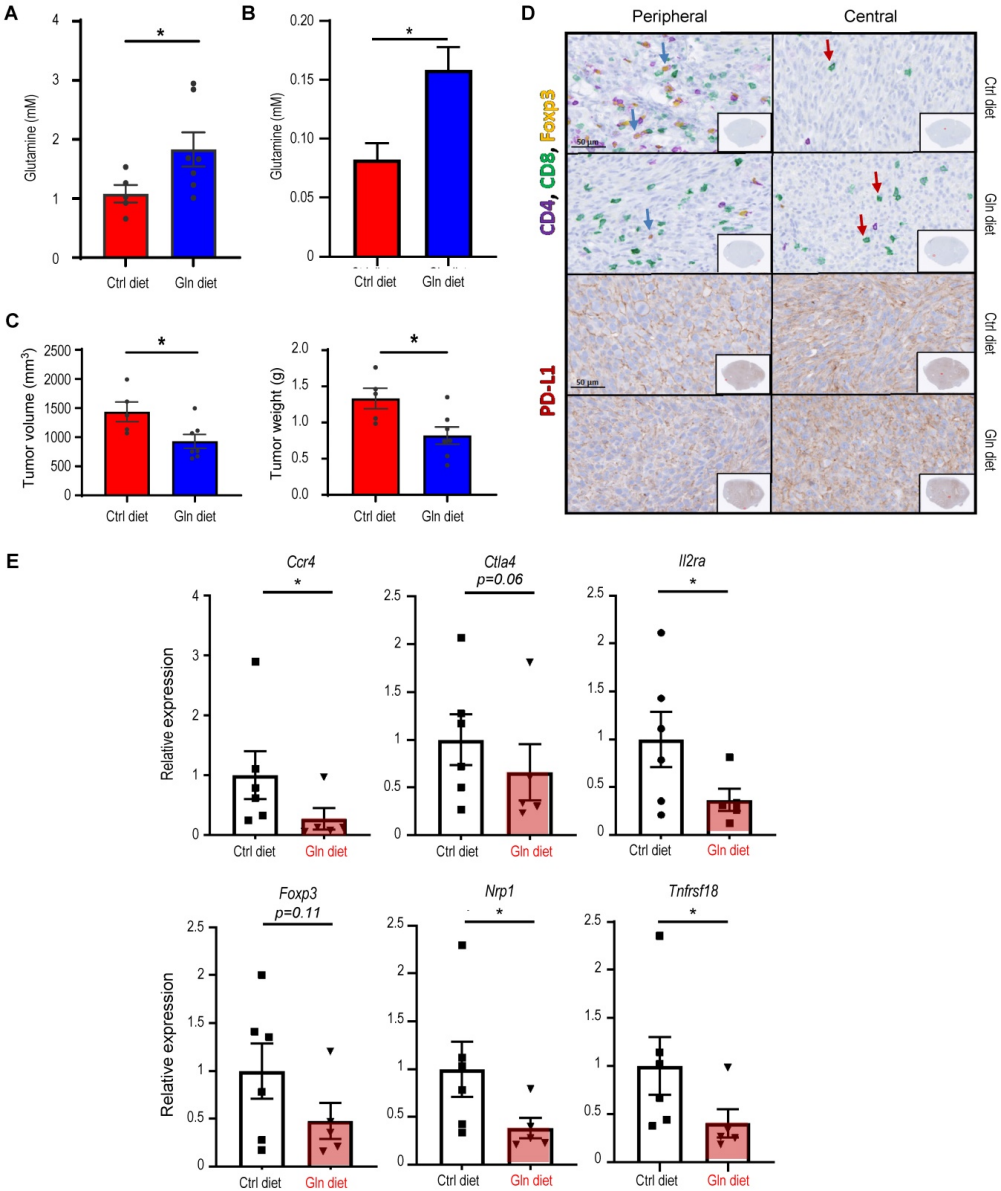
** Dietary glutamine supplementation reduces MST tumor burden with increased CD8^+^ cell infiltration**. (**A**, **B**) 28 days of dietary glutamine supplementation increases glutamine concentration in plasma of naïve mice (**A**) and in SMG of tumor-bearing mice (**B**) (n ≥ 5 in each cohort). (**C, D**) Mice with orthotopic MST implantation were fed with control (Ctrl diet, n = 5) and glutamine-supplemented diet (Gln diet, n = 7), respectively, for 21 days prior to tumor implantation and for another 21 days after implantation, prior to euthanasia. Tumor volume (*left*) and wet SMG weight (*right*) at Day 21 post-tumor implantation are shown (**C**), and representative micrographs of indicated IHC stains of the orthotopic MST tumors are shown (**D**). (*Upper 4 panels*) IHC staining for CD4, CD8 and Foxp3 cells in FFPE tumor sections. Green arrows indicate Foxp3-positive (yellow nuclei staining) cells. Red arrows indicate CD8^+^-positive (green membrane staining) cells. Inlets are a low-power overview (red boxes indicate relative spatial location of enlarged views (scale bar: 50 μm). Peripheral (*left*) is considered < 500 μm from the edge, central/core (non-necrotic region) > 500 μm from the edge. (*Lower 4 panels*) IHC staining of tumor sections using PD-L1 antibody (red membrane staining). Images are representatives of ≥ 5 biological replicates. Nuclei were stained with hematoxylin (blue). Data are shown as mean ± SD; *: *p* < 0.05 by Welch* t* test. (**E**) Dietary glutamine supplementation promotes an immunosuppressive gene signature. Mice with orthotopic MST implantation (50,000 MST cells/mouse) were fed with control (Ctrl diet, n = 6) and high glutamine (Gln diet, n = 5) diets prior to tumor harvesting. mRNA levels of indicated genes were determined by qRT-PCR; data are representative of three independent experiments. *: *p* < 0.05; Student's *t* test.

Next, we evaluated the effect of dietary glutamine supplementation on MST tumor growth. We used isocaloric diet with 20% additional glutamine (high glutamine diet) compared to control diet as reported by Ishak Gabra 43. Supplementation of glutamine in the diet significantly increases the plasma concentration of glutamine in naïve *Atg5*^+/+^ mice (**Figure 6A**). Furthermore, comparing to the *Atg5*^+/+^ mice fed with control diet, tumor glutamine level was elevated in the *Atg5*^+/+^ mice fed with high glutamine diet (**Figure 6B**). These immunocompetent *Atg5*^+/+^ mice fed with high glutamine diet developed significantly smaller tumors after orthotopic tumor implantation (**Figure 6C**). H&E staining of the excised tumors from mice fed with high glutamine diet showed areas of residual normal glandular parenchyma (**Figure S6).** IHC staining revealed that infiltrating cytotoxic CD8^+^ T cells were more notably abundant with overall less Foxp3^+^ Tregs detected in tumor sections from high glutamine-fed mice (**Figure 6D**, *upper 4 panels*). Notably, tumor PD-LI signals were moderately strong without clear spatial distribution or affected by high glutamine diet (**Figure 6D**, *lower 4 panels*). Presumably, the increase in tumor-infiltrating CD8^+^ T cells is caused by the reduction of Tregs. To test this possibility, we performed studies and showed that tumors harvested from high glutamine diet-fed mice exhibited lower levels of Treg expressed or function-associated RNA transcripts, such as *Foxp3* (key transcription factor in Tregs), *Tnfrsf18* (cell surface receptor in Tregs), *Ctla4* and *Nrp1* (both involved in immunosuppressive mechanisms by Tregs), *Il2ra* (receptor for IL-2 which is an essential cytokine in differentiating Tregs) and *Ccr4* (expression in Treg may allow their migration toward APCs and activated T cells leading to inhibition of APC function or suppression of responding T cells), when compared to tumors from control-diet fed mice (**Figure 6E**). These results indicate that high glutamine diet leads to lessened expression of immunosuppressive gene signature in Tregs. Altogether, dietary intake of glutamine may effectively increase the concentration of glutamine in TME to suppress Treg differentiation, mimicking an autophagy-compromised TME (**Table 1**).

## Discussion

Immune suppression and escape are increasingly recognized as critical traits of malignancy 58. During cancer progression, autophagy may represent an important pathway for immune escape, while also promoting the malignant phenotype of cancer cells 59, 60. The increasing interests in the role of compromised autophagy, in addition to undermining tumorigenesis, in controlling immune tolerance and bolstering tumor rejection 61, prompted us to develop a syngeneic orthotopic mouse tumor model for evaluating host autophagy capacity on MST progression. This novel syngeneic tumor model enables us to show for the first time that host ATG5-dependent autophagy promotes tumor progression by suppressing the antitumor immune response, independently of the autophagy genotypes of donor tumor cells. In other words, the attenuated host autophagy capacity ultimately results in spontaneous tumor regression and improved survival of tumor-bearing mice through an “antitumor” TME.

Mukha et al. have shown that inhibition of autophagy by genetic knockdown of the *ATG5* gene significantly increased the radiosensitizing effect of glutamine deprivation, suggesting that in response to glutamine deprivation, cancer cells activate ATG5-mediated autophagy as a survival mechanism to overcome nutrient stress 62, 63. Further, a pilot randomized trial showed that oral glutamine inhibited the autophagic response in cancer patients treated with radiotherapy 64. Beyond that, autophagy also plays a key role in the function and development of neutrophils, macrophages, NK cells, T cells and B cells and dendritic cells 65, key components of TME. In general, the relationship between autophagy and immune system is complex, and there is no consensus on the role autophagy plays in antitumor immunity. Our data suggest at least two of these populations, T lymphocytes and macrophages, are affected by attenuated host autophagy within TME. The improved antitumor TME in *Atg5^flox/flox^* mice is consistent with reports from recent studies that implicate autophagy in immune evasion which may restrain antitumor immunity 66, 67. For example, Cunha et al. reported that the growth of subcutaneously engrafted murine melanoma is suppressed in ATG5-compromised mice by M1-polarized TAMs and increased type I IFN production 68. Further, autophagy promotes tumor immune tolerance by enabling Treg function and limiting expression of IFN and CD8^+^ T cell response which in turn enables tumor growth 69. Likewise, blocking hypoxia-induced autophagy in tumors restores cytotoxic T cell activity and promotes regression in lung cancer 36. Additionally, loss of host autophagy increases the level of circulating pro-inflammatory cytokines and promotes T cell infiltration in tumors with high tumor mutational burden 69. Consistent with these reports, we showed that autophagy is a critical immune-suppressing factor that regulates the infiltration and activity of cytotoxic CD8^+^ T cells. We found that attenuation, even not complete depletion, of ATG5 abundance alone is sufficient to increase IFN-γ expression by IFN-γ producing cells at both early and later tumor stages in *Atg5^flox/flox^* mice. Tumor-infiltrating CD8^+^ T cells are a useful prognostic parameter in various cancers 70. Indeed, CD8^+^ T cells are restrained due to long-lasting interactions with TAMs, whereas depletion of TAMs restores T cell migration and infiltration into tumor islets 71. Consistently, we found that there are more macrophages infiltrating the tumors in *Atg5*^+/+^ mice, which may impede migration of CD8^+^ T cells into the TME.

Herein, we also report glutamine to be an immunometabolic regulator in SMGs that links compromised autophagy to immunosuppressive Foxp3^+^ Tregs. Tregs play a crucial role in the prevention of antitumor immunity by suppressing the activation and differentiation of CD4^+^ helper T cells and CD8^+^ cytotoxic T lymphocytes 72. In this study, we found that increased Tregs infiltrate was accompanied with low CD8^+^IFN-γ^+^ infiltrate in SMGs and spleens in tumor-bearing *Atg5*^+/+^ mice at Day 25. Higher Treg infiltrate within SMG TME of tumor-bearing *Atg5*^+/+^ mice would inhibit CD8^+^ cytotoxic T cells, leading to a tumor progression phenotype. Moreover, we found that glutamine supplementation inhibited the skewing of naïve T cells isolated from the spleen into Tregs. In concordance with our previous studies 40, the difference in intratumoral glutamine level was prominent between SMGs of *Atg5^+/+^* and *Atg5^flox/flox^* recipient mice (**Figure 5C**). Glutamine is a non-essential, but the most abundant amino acid in the body 73. It participates in central metabolic processes by acting as an energy substrate for the tricarboxylic acid cycle and a nitrogen donor in several pathways including purine/pyrimidine synthesis, nicotinamide adenine dinucleotide metabolism, and the urea cycle 74, 75. Several mechanisms have been suggested to link glutamine and antitumor immunity. In macrophages and T cells, it has been reported to be mediated via shifts in energy utilization (*i.e.*, the balance between glycolysis and glutaminolysis), which alters the levels of intermediary metabolites such as αKG 76.

α-ketoglutarate produced by glutaminolysis promotes mammalian target of rapamycin complex 1 (mTORC1) localization to the lysosome via regulating RAGB GTP loading and consequently RAG activity 77. The upregulation of glutaminolysis provides energetic advantages to proliferating cancer cells. Glutaminolysis inhibition upregulates ATF4 expression to activate pro-survival autophagy through mTORC1 inactivation 78. Inhibition of glutaminolysis leads to an inhibition of mTORC1, it would also promote the activation of autophagy, which is crucial for the survival of cancer cells, suggesting that simultaneous inhibition of both glutaminolysis and autophagy could potentially synergize antitumor effect 79. Moreover, Han et al. found that Fas apoptotic inhibitory molecule could promote the tetramer formation and stabilization of glutaminase C, thereby increasing the production of αKG, and leading to the activation of mTOR in lung adenocarcinoma 80. In addition, a report by Tran et al. showed that glutamine-αKG axis suppresses Wnt signaling and promotes cellular differentiation, thereby restricting tumor growth in colorectal cancer 81. Furthermore, αKG-dependent demethylation is a critical regulatory step in T cell activation and differentiation and macrophage polarization 82-84. In TME, mTOR regulates function and differentiation of Tregs, and hence modulates immune responses 85-87. In contrast, inhibition of mTORC1 promotes differentiation toward a memory phenotype 88 but not into to an effector phenotype 89 in CD8^+^ T cells. Herein, we demonstrate that high glutamine levels reduced CD4^+^CD25^+^Foxp3^+^ Treg cell population (**Figure 5E-F**). Here, we established a causal link between dietary glutamine supplementation and antitumor immunity in mouse MST was established.

Nutritional stress is used by cancer cells to generate an immunosuppressive microenvironment to impact the function of tumor-infiltrating lymphocytes 13, 90-92. Within tumors, intratumor nutrient level is determined by the net balance of host blood supply, autophagy, and the net competition between tumor cells and other TME residents 90. In addition, the increased metabolic demands of tumor cells and activated T lymphocytes may introduce competition for glutamine within the TME 93, creating a scenario in which tumor cells out-compete T cells for local glutamine and thereby alter the characteristics of the tumor-infiltrating lymphocytes. Thus, in this scenario the glutamine consumption would both promote proliferation and survival of tumor cells and limit the capacity for T cell-mediated antitumor immunity simultaneously, similar to observations with arginine 90. Accordingly, we postulated that glutamine-consuming tumors, such as MSTs, might benefit therapeutically from dietary glutamine supplementation, improving antitumor T cell responses by reversing a tumor “glutamine grab” phenomenon. However, other immune cells may also be affected by dietary glutamine supplementation. For example, the production of αKG via glutaminolysis is important for activation of M2-like macrophages 84. Thus, although our findings of improved intratumoral T cell effector functions likely result from the increased glutamine availability to suppress Tregs, the potential remains for additional factors that can impact the immune system such as inhibition of suppressive microenvironments by M2-like macrophages. It is also possible that different residents within TME use distinct nutrients according to their own unique metabolic programs. The dietary glutamine supplementation enables a metabolic signaling pathway that suppress the function of some immune system T cells to promote others. The concept of preventing the “glutamine steal” by tumor cells as a treatment strategy may be applicable beyond MSTs as multiple types of tumors are also considered to be glutamine-addicted. It is possible that this phenomenon is playing out in other cancers as well.

## Conclusions

In summary, we found an elevated expression of basal proinflammatory cytokines in the SMGs of naïve *Atg5^flox/flox^* mice with attenuated autophagy. Subsequently, we developed a syngeneic orthotopic MST tumor model in *Atg5^+/+^* and *Atg5^flox/flox^* mice and revealed that *Atg5^flox/flox^* mice suppressed orthotopically allografted MST cells. Together with reduced growth of tumors, there was an enhanced antitumor immune response demonstrated by reduction of both M1 and M2 macrophages, increased infiltration of CD8^+^ T cells, elevated IFN-γ production, as well as decreased inhibitory Tregs within TME and spleens of recipient mice. Mechanistically, attenuated autophagy led to increased levels of glutamine within SMGs which in turn would promote the inflammatory T cells while inhibiting the generation of Tregs in tumor-bearing *Atg5^flox/flox^* mice. In addition, dietary glutamine supplementation, mimicking attenuated autophagy, retarded tumor expansion in *Atg5^+/+^* mice.

## Supplementary Material

Supplementary figures and table.

## Figures and Tables

**Table 1:** Immune cell profile in autophagy-deficient versus autophagy-sufficient tumor microenvironments at two selected endpoints, Day 14 and Day 25, post-tumor implantation. Data summarized is based on flow cytometry analyses from **Figure 2, Figure 3, Figure 4, Figure 5** and **Figure S2**.

Host genotypes	*Atg5^flox/flox^* versus* Atg5^+/+^*	
Time points	Day 14	Day 25
NK cells	-	-
Neutrophils	-	-
CD4^+^ T cells	-	-
CD8^+^ T cells	↑	↑
CD4^+^IFN-γ^+^	-	↑
CD8^+^IFN-γ^+^	-	↑
TAMs (M1 and M2)	-	↓
Tregs	↓	↓

## Abbreviations

αKG: α-ketoglutarate
ATG3: autophagy-related 3
ATG5: autophagy-related 5
ATG7: autophagy-related 7
ATG12: autophagy-related 12
CD25: cluster of differentiation 25
CD28: cluster of differentiation 28
CD3: cluster of differentiation 3
CD4: cluster of differentiation 4
CD8: cluster of differentiation 8
FOXP3: forkhead box P3
IFN-γ: interferon gamma
IL1: interleukin-1
IL2: interleukin-2
IL6: interleukin-6
Treg: regulatory T cell
KRAS: kirsten rat sarcoma viral oncogene homolog
LPS: lipopolysaccharide
MST: malignant salivary gland tumor
mTORC1: mammalian target of rapamycin complex 1
NK: natural killer cell
P21: cyclin dependent kinase inhibitor 1A
RAGB: Ras-related GTP-binding protein B
SDC: salivary duct carcinoma
SMG: submandibular gland
TAM: tumor-associated macrophage
TGFB: transforming growth factor beta
Th1: type 1 T helper cell
Th2: type 2 T helper cell
TME: tumor microenvironment
TNFA: tumor necrosis factor alpha
Treg: regulatory T cell
