# Supplementary material for: Glutamine is essential for overcoming the immunosuppressive microenvironment in malignant salivary gland tumors
Source: Theranostics. 2022 Aug 8;12(13):6038–56. doi: 10.7150/thno.73896 (PMC9373812; doi:10.7150/thno.73896)
Supplement: Supplementary file 1 — Supplementary figures and table. [file thnov12p6038s1.pdf]

Glutamine is essential for overcoming the immunosuppressive microenvironment in malignant salivary gland tumors

-Supplementary Figures and Table

Shuting Cao[a], Yu-Wen Hung[a, #], Yi-Chang Wang[a, #], Yiyin Chung[a, #], Yue Qi [a, #], Ching Ouyang[b], Xiancai Zhong[c], Weidong Hu[c], Alaysia Coblentz[a], Ellie Maghami[d], Zuoming Sun[c, e], H. Helen Lin[a], and David K. Ann[a, e, *]

[a]Department of Diabetes Complications and Metabolism, Arthur Riggs Diabetes and Metabolism Research Institute, Beckman Research Institute, City of Hope, Duarte, CA, 91010, USA

[b]Department of Computational and Quantitative Medicine, Beckman Research Institute, City of Hope Comprehensive Cancer Center, Duarte, CA 91010, USA

[c]Department of Immunology and Theranostics, Beckman Research Institute, City of Hope Comprehensive Cancer Center, Duarte, CA 91010, USA

[d]Division of Head and Neck Surgery, City of Hope National Medical Center, Duarte, CA 91010, USA

[e]Irell & Manella Graduate School of Biological Sciences, Beckman Research Institute, City of Hope Comprehensive Cancer Center, Duarte, CA 91010, USA

[#]Contribute equally
*To whom all correspondence should be addressed:
David K. Ann, Ph.D.
Beckman Research Institute
City of Hope Comprehensive Cancer Center
Duarte, CA 91010-3000
Tel: 626-218-6586
E-Mail: dann@coh.org

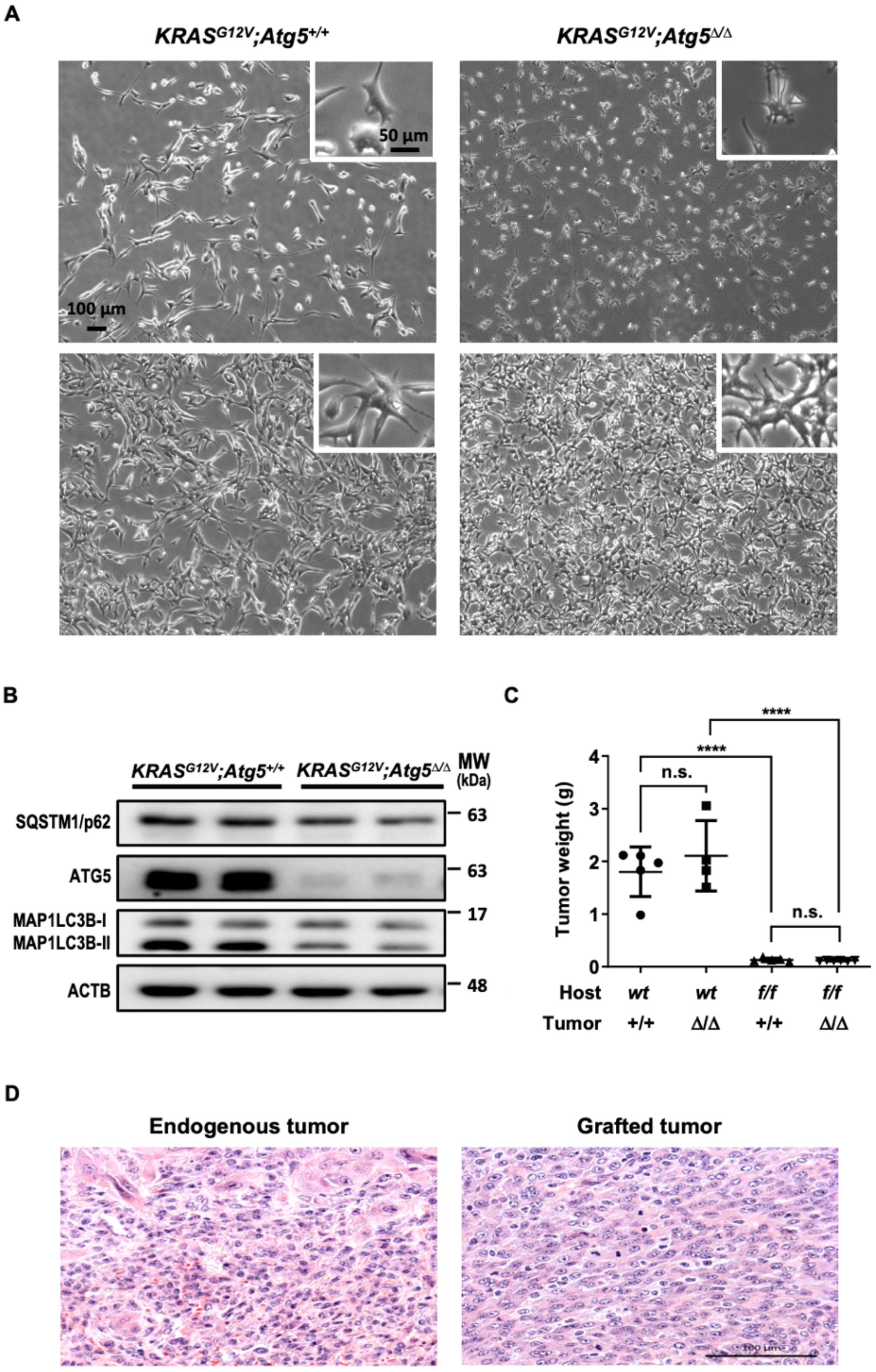
A
KRASG12V;Atg5+/+
KRASG12V;Atg5Δ/Δ
50 µm
100 µm
B
KRASG12V;Atg5+/+
KRASG12V;Atg5Δ/Δ
MW (kDa)
SQSTM1/p62
ATG5
MAP1LC3B-I
MAP1LC3B-II
ACTB
63
63
17
48
C
Tumor weight (g)
n.s.
****
****
n.s.
Host wt wt f/f f/f
Tumor +/+ Δ/Δ +/+ Δ/Δ
D
Endogenous tumor
Grafted tumor

Figure S1. Characterization or primary salivary carcinoma cells isolated from different hosts.

Primary tumor cells were isolated from tamoxifen-induced malignant salivary tumors. (**A**) Cell morphology of *KRAS*^{G12V};*Atg5*$^{+/+}$ and *KRAS*^{G12V};*Atg5*$^{\Delta/\Delta}$ malignant salivary tumor cells grown at low (top panels) and high (bottom panels) confluency. Enlarged images are also shown (inserts). (**B**) Representative western blots, in which total lysates from *KRAS*^{G12V};*Atg5*$^{+/+}$ and *KRAS*^{G12V};*Atg5*$^{\Delta/\Delta}$ malignant salivary tumor cells were stained with indicated antibodies. Two each of independent primary salivary tumor cells were isolated from tumor-bearing ATG5-wild type and ATG5-knockout mice, respectively, are shown. (**C**) Malignant salivary tumor cells *KRAS*^{G12V};*Atg5*$^{+/+}$ (+/+) and *KRAS*^{G12V};*Atg5*$^{\Delta/\Delta}$ (Δ/Δ) were orthotopically implanted into SMG of wild type (*wt*) and *Atg5*$^{flox/flox}$ (*f/f)* host mice. Tumor weights were measured on Day 25 post-implantation. n ≥ 4. n.s., not significant; ****: $p < 0.0001$; Student's *t*-test, 2-tailed, unpaired. (**D**) Comparison between H&E stain of tumor sections of endogenous tamoxifen-induced salivary tumors and orthotopically implanted tumors. Scale bar; 100 μm.

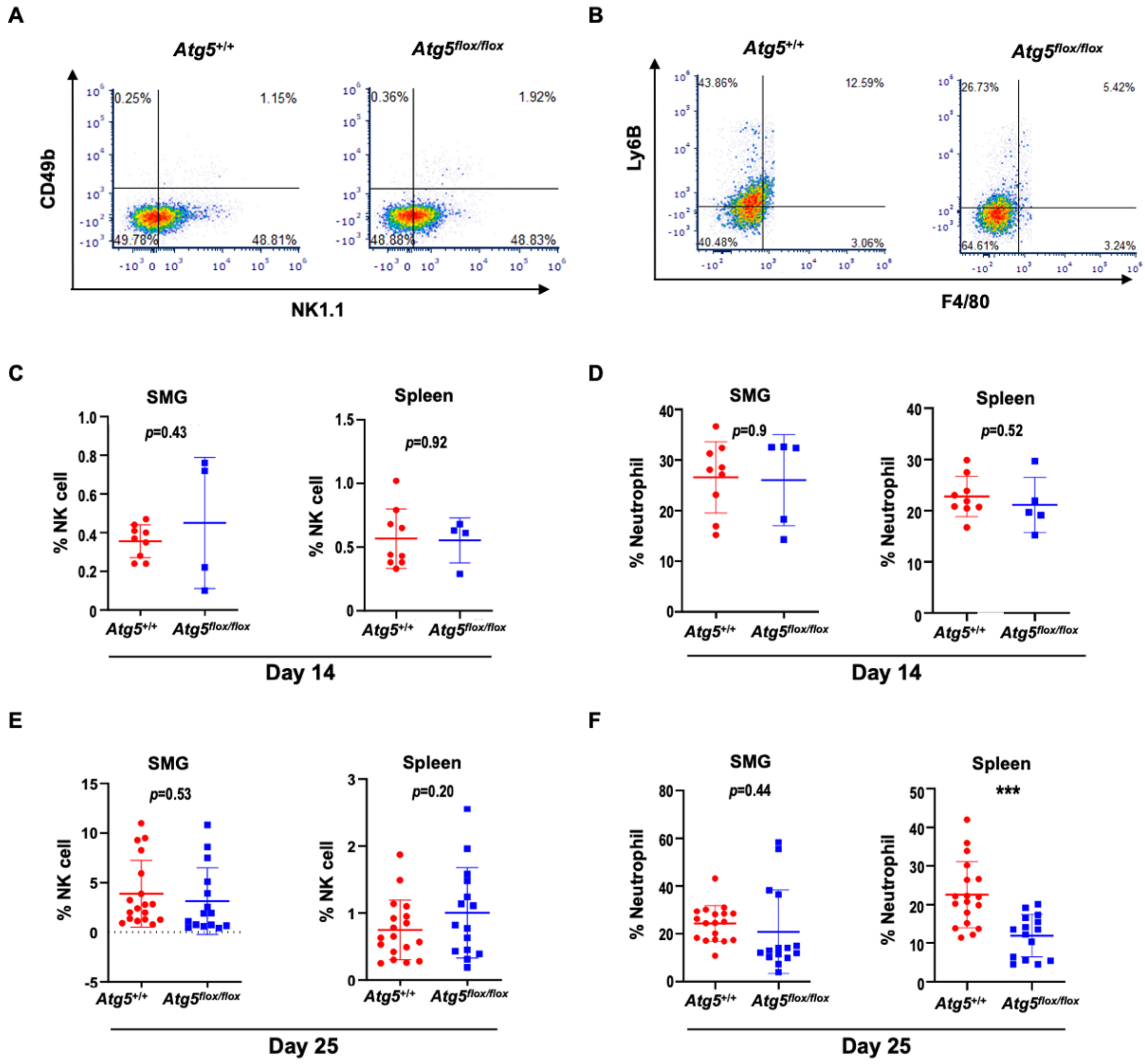

Figure S2. Natural killer cells and neutrophils population in SMG TME.

(**A**, **B**) Representative flow cytometry showing $CD11b^+CD49b^+NK1.1^+$ NK cells (**A**), and $CD11b^+F4/80^-Ly6B^+$ neutrophils (**B**) isolated from live splenocytes of Day 25 tumor-bearing *Atg5*$^{+/+}$ and *Atg5*$^{flox/flox}$ mice. (**C, E**) Flow cytometry analyses showing the percentage of NK cells ($CD11b^+CD49b^+NK1.1^+$) in alive SMG cells and splenocytes from Day 14 (**C**) and Day 25 (**E**) tumor-bearing *Atg5*$^{+/+}$ and *Atg5*$^{flox/flox}$ mice. (**D, F**) Flow cytometry analyses showing the percentage of neutrophils ($CD11b^+F4/80^-Ly6B^+$) in alive SMG cells and splenocytes from Day 14 (**D**) and Day 25 (**F**) tumor-bearing *Atg5*$^{+/+}$ and *Atg5*$^{flox/flox}$ mice. Day 14 tumor-bearing mice: *Atg5*$^{+/+}$: n = 9; *Atg5*$^{flox/flox}$: n = 5. Day 25 tumor-bearing mice: *Atg5*$^{+/+}$: n = 18; *Atg5*$^{flox/flox}$: n = 15. Data are shown as mean ± SD; ***: $p < 0.001$; Student's *t*-test, 2-tailed, unpaired.

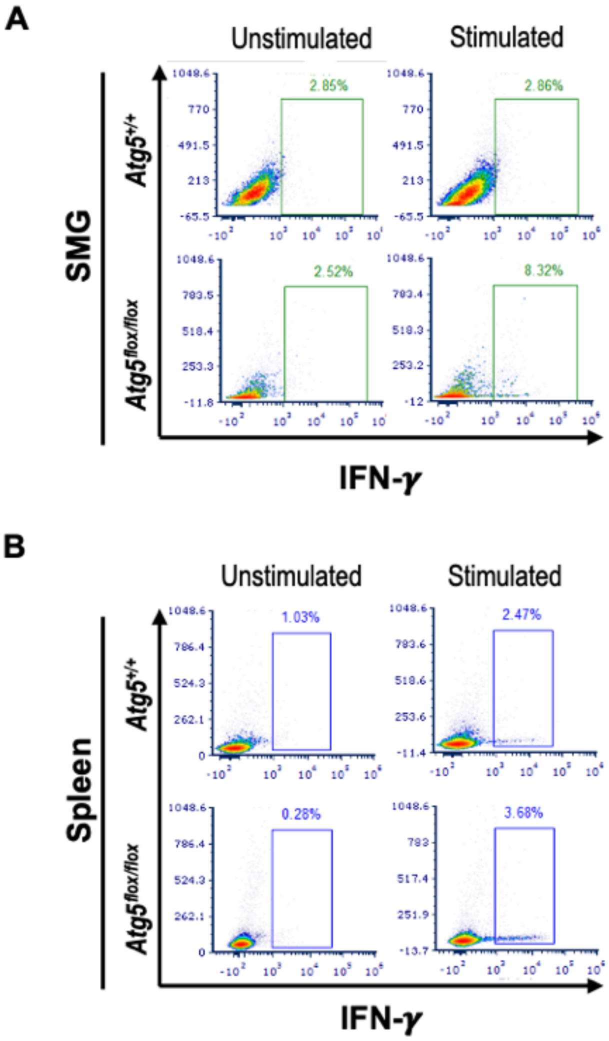

Figure S3. Flow cytometry analysis of IFN-γ-producing cells in SMGs and spleens of tumor-bearing mice.

Representative flow cytometry showing IFN-γ-producing cells in single cell suspensions of the alive SMG cells (**A**) and splenocytes (**B**) of tumor-bearing *Atg5*$^{+/+}$ and *Atg5*$^{flox/flox}$ mice following PMA/ionomycin/Golgiplug stimulation for 4h (Stimulated) or unstimulated control (Unstimulated).

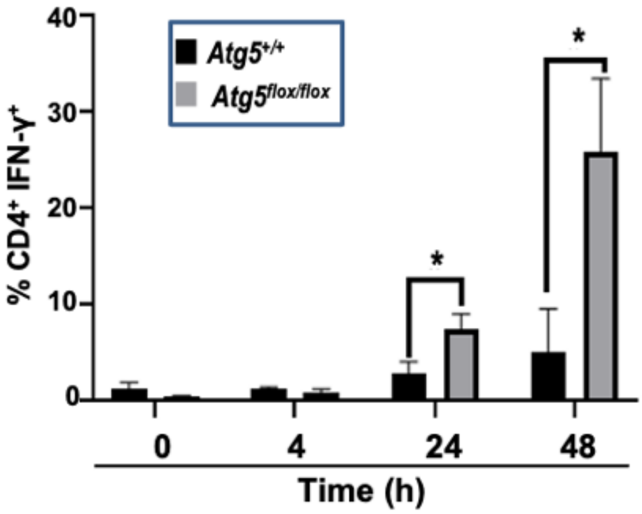

Figure S4. Glutamine promotes IFN-γ$^+$ production by CD4$^+$ T cells from *Atg5$^{flox/flox}$* mice. CD4$^+$ T cells were isolated from splenocytes of naïve *Atg5$^{+/+}$* and *Atg5$^{flox/flox}$* mice and cultured in glutamine-free Treg polarization medium for 3 days. Afterwards, cells were cultured in fresh Treg polarization medium supplemented with glutamine (2 mM) for the indicated time periods (0, 4, 24 and 48 h), and then stimulated with PMA/ionomycin/ Golgiplug for 4 h. Percentages of IFN-γ-positive CD4$^+$ cells were determined by flow cytometry analyses (n = 3). Data are presented in bar graph shown as Mean ± SEM. *p* value was calculated by *t* test (unpaired, two tailed). *: $p < 0.05$.

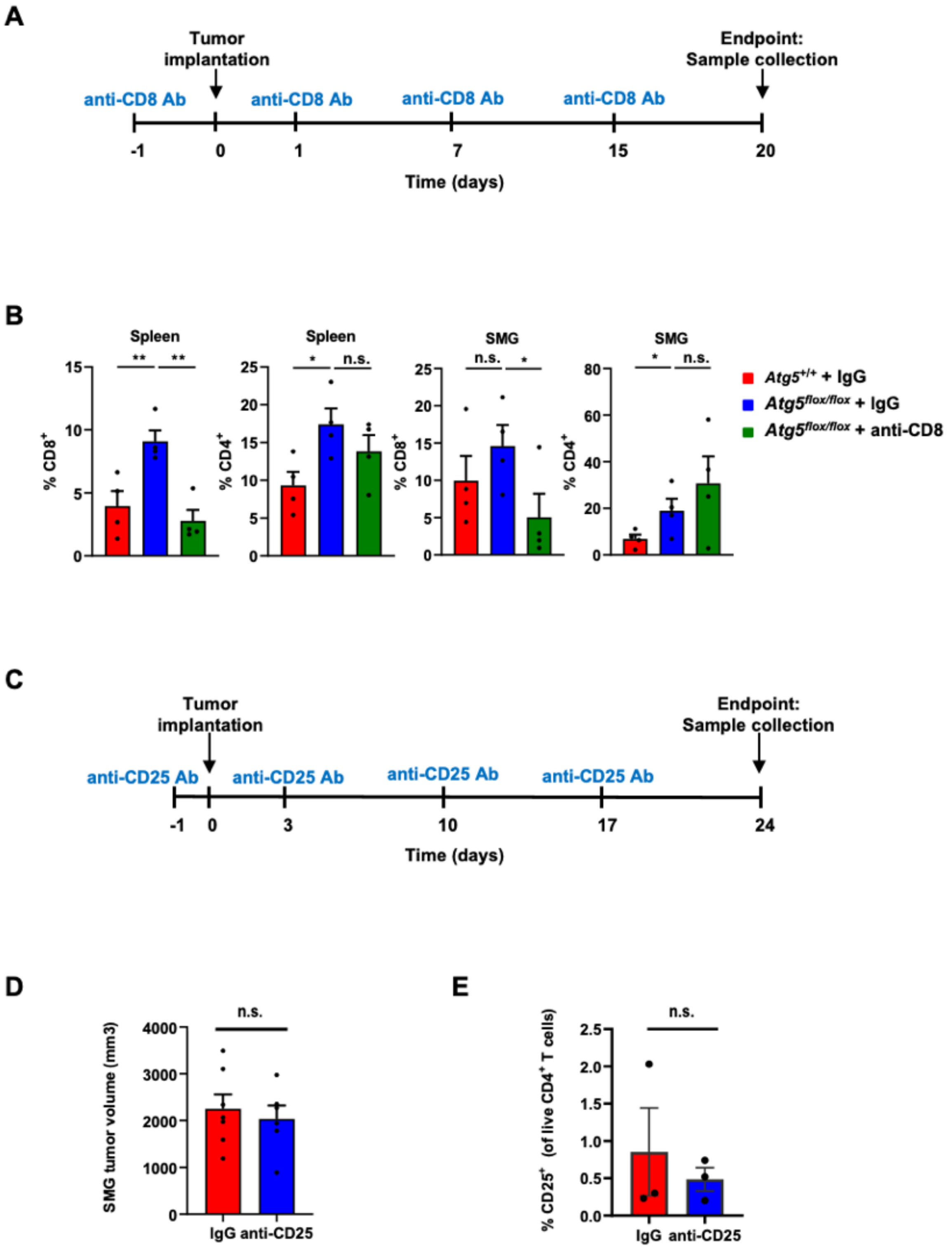

Figure S5. CD8$^+$ T cells play a major role in antitumor activity.

(**A**) Diagram showing time schedule when *Atg5*$^{+/+}$ and *Atg5*$^{flox/flox}$ mice were inoculated with MST cells orthotopically and received an anti-CD8 or IgG antibody (0.2 mg/mouse; *i.p.*), as indicated. Tumors and spleens were collected at Day 20. (**B**) Flow cytometric analysis of CD8$^+$

and CD4$^+$ T cell subsets. Local administration of clone 2.43 reduced population of CD8$^+$ T cells in spleen and SMG of *Atg5*$^{flox/flox}$ mice at 5 days after the last treatment. All graphs were CD8$^+$- and CD4$^+$-gated. Percentages of CD8$^+$ and CD4$^+$ cells in spleen (*left 2 panels*) and tumor-bearing SMG (*right 2 panels*) are shown. Data are presented in bar graph shown as Mean ± SEM. *p* value was calculated by Welch's *t* test, one tailed, unpaired. *Atg5*$^{+/+}$ + IgG: n = 8; *Atg5*$^{flox/flox}$ + IgG: n = 7; *Atg5*$^{flox/flox}$ + anti-CD8: n = 10; *: $p < 0.05$; **: $p < 0.01$; *n.s.*, not significant. (**C**) Diagram showing time schedule when *Atg5*$^{+/+}$ mice were inoculated with MST cells orthotopically and received an anti-CD25 or IgG antibody (0.25 mg/mouse; *i.p.*), as indicated. Tumors were harvested at Day 24. (**D**) The depletion of CD25$^+$ T cells by clone PC-61.5.3 had a very modest effect on reducing SMG tumor burden. (**E**) Local administration of clone PC-61.5.3 has limited effect on the population of CD25$^+$ T cells at 7 days after the last treatment in SMG of *Atg5*$^{+/+}$ mice. Graphs were CD25$^+$-gated. Percentage of CD25$^+$ (of live CD4$^+$ T cells) in tumor-bearing SMG is shown. Data are presented in bar graph shown as Mean ± SEM. *p* value was calculated by Welch's *t* test, one tailed, unpaired. *Atg5*$^{+/+}$ + IgG: n = 7; *Atg5*$^{+/+}$ + anti-CD25: n = 6; *n.s.*, not significant.

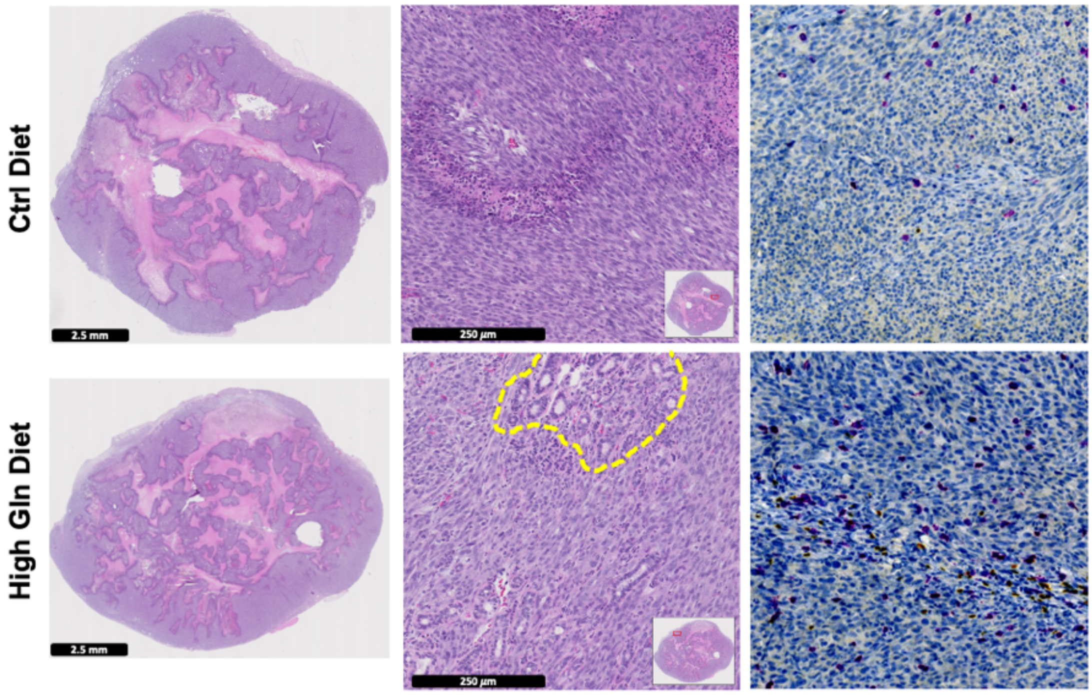

Figure S6. High glutamine diet reduces MST tumor burden with increased CD8+ T cell infiltration.

Gross view (*left*) and enlarged view (*middle*) of H&E-stained tumor sections from two mouse cohorts, each fed with either a control (Ctrl) diet or high glutamine diet, respectively. Areas of residual salivary gland, *enclosed by a yellow dashed line,* can be seen in tumor from mice on a high glutamine diet. IHC staining with an anti-CD8 antibody shows increased tumor-infiltrating CD8 T cells (*purple in color*; *right*) in tumors from *Atg5*$^{+/+}$ mice fed with high-glutamine diet.

Table S1: Primer sets for RT-PCR analyses.

Gene	Forward primer	Reverse primer
Cdkn1a	5’- AGG AGC AAA GTG TGC CGT TG -3’	5’- CGA AGT CAA AGT TCC ACC GTT C -3’
Ccr4	5'- GGA AGG TAT CAA GGC ATT TGG G -3'	5'- GTA CAC GTC CGT CAT GGA CTT -3'
Ctla4	5'- TTT TGT AGC CCT GCT CAC TCT -3'	5'- CTG AAG GTT GGG TCA CCT GTA -3'
Foxp3	5’- ACC ATT GGT TTA CTC GCA TGT -3’	5’- TCC ACT CGC ACA AAG CAC TT -3’
Gapdh	5’- CCC CTT CAT TGA CCT CAA CTA -3’	5’- CTC CTG GAA GAT GGT GAT GG -3’
Ifng	5’- CTT GAA CCC TGT CGT ATG CTG G -3’	5’- TTG GTG CAG GAA TCA GTC CAG G -3’
Il1a	5’- TGT TGC TGA AGG AGT TGC CAG -3’	5’- CCC GAC TTT GTT CTT TGG TGG -3’
Il1b	5’- TGG ACC TTC CAG GAT GAG GAC A -3’	5’- GTT CAT CTC GGA GCC TGT AGT G -3’
Il2ra	5'- CGG GCA GAA CTG TGT CTG TA -3'	5'- GTT GCT GCT CCA GGA GTT TC -3'
Il6	5’- TAC CAC TTC ACA AGT CGG AGG C -3’	5’- CTG CAA GTG CAT CAT CGT TGT TC -3’
Nrp1	5'- GAC AAA TGT GGC GGG ACC ATA -3'	5'- TGG ATT AGC CAT TCA CAC TTC TC -3'
Tnf	5’- GGT GCC TAT GTC TCA GCC TCT T -3’	5’- GCC ATA GAA CTG ATG AGA GGG AG -3’
Tnfrsf18	5'- GCC ATG CTG TAT GGA GTC TCG -3'	5'- CCA CTT CCG TTC TGA ACC TTG -3'
Rps18	5’- TTC CAG CAC ATT TTG CGA GTA -3’	5’- CAC GCC CTT AAT GGC AGT GAT -3’
